# Efficient Charge Transfer in TiOPc/MoS_2_ Heterostructure for Dynamically Enhanced SERS Sensing and Photocatalysis

**DOI:** 10.3390/molecules31101644

**Published:** 2026-05-13

**Authors:** Muhammad Saleem, Min Li, Shuai Qiu, Muhammad Zahid, Min Li, Chengju Guo, Abdur Rahim, Yuzhi Song, Mei Liu

**Affiliations:** School of Physics and Optoelectronics, Shandong Normal University, Jinan 250358, China

**Keywords:** SERS, organic/inorganic heterostructure, efficient charge transfer, molybdenum disulfide, titanium oxophthalocyanine

## Abstract

Surface-enhanced Raman scattering (SERS) offers exceptional sensitivity for trace contaminant detection; conventional noble-metal substrates suffer from high cost, signal irreproducibility, and poor chemical stability. While semiconductor alternatives are promising, their performance is fundamentally limited by sluggish interfacial charge-transfer kinetics under static band alignment. To overcome these limitations, we introduced a new strategy centred on a high carrier generation rate (HCGR). By integrating TiOPc, a material that exhibits strong Ti–O bond polarisation and a high HCGR, with atomically thin MoS_2_, we constructed a hybrid platform that drives efficient charge transfer via HCGR-enabled kinetic pumping, surpassing traditional thermodynamic band engineering. This HCGR-driven efficient CT mechanism primarily amplifies SERS through enhanced chemical mechanisms (CM) with minor electromagnetic contributions, achieving an enhancement factor (EF) of 10^7^. The platform can detect methylene blue (MB) and rhodamine 6G (R6G) at concentrations as low as 10^−14^ M and 10^−13^ M, respectively, demonstrating excellent repeatability (RSD = 7.2%) and stability over 60 days. Additionally, efficient CT accelerated MB photodegradation under UV light, achieving complete decomposition within 80 min. The practical applicability of the platform is evidenced by detecting Hg^2+^ (LOD: 10^−11^ M) and malachite green in tap/lake water (LODs: 10^−12^ M/10^−10^ M). This work establishes HCGR-driven efficient CT as the next generation of semiconductor SERS platforms. It provides a scalable route toward low-cost, reusable sensors for real-time, in situ monitoring of industrial effluents and the dynamic pollutant degradation of pollutants in environmental monitoring.

## 1. Introduction

Surface-enhanced Raman scattering (SERS) is a powerful analytical technique distinguished by its high sensitivity, molecular specificity, and rapid response, enabling trace-level detection of biological and chemical contaminants [1,2,3]. Its capacity for multi-analytes has established SERS as a suitable tool for real-time monitoring in food safety, environmental monitoring, and biomedical diagnostics [2,4,5,6]. Signal enhancement in SERS is governed by two principles: electromagnetic enhancement (EM), which originates from localised surface plasmon resonance amplifying local electromagnetic fields, and chemical enhancement (CM), which arises from charge transfer interactions between the substrate and adsorbed molecules [6,7]. Noble metal-based SERS substrates achieve high enhancement factors (EF) (10^8^–10^9^); their practical application is limited by inherent drawbacks, including poor selectivity, high cost, chemical instability, and irreproducible nanostructure that results in signal fluctuations [7,8,9]. Semiconductor-based SERS substrate alternatives offer promise but suffer from inherently sluggish charge-transfer kinetics at heterointerfaces. This fundamental bottleneck, often misattributed solely to “low sensitivity,” stems from inefficient carrier injection and recombination losses under static band alignment, severely limiting SERS performance [10,11,12].

Conventional semiconductor SERS design relies predominantly on static band engineering, where heterostructures such as MoS_2_/WS_2_ are tailored for thermodynamically favourable band offsets to promote charge separation [13]. However, thermodynamic viability does not guarantee kinetic efficiency. Effective CM requires continuous, high-flux photogenerated carriers to rapidly populate molecule-coupled charge transfer (CT) states under optical excitation. Therefore, even optimally aligned heterostructures may exhibit weak or unstable SERS signals if carrier generation is insufficient or recombination outpaces interfacial injection, depleting surface carriers and diminishing CT amplification [14,15,16,17,18].

To overcome these challenges, we proposed a new strategy centred on High Carrier-Generation Rate (HCGR) as a “kinetic pump”. This approach transcends static band engineering by sustaining non-equilibrium carrier populations to enable efficient charge transfer (ECT) under illumination. Titanium oxophthalocyanine (TiOPc) serves as an ideal HCGR component due to the strong polarisation of its Ti–O bond within the porphyrin macrocycle, which facilitates efficient exciton dissociation and maximises carrier flux to the heterointerface [19]. TiOPc exhibits HCGR near 10^22^ cm^−3^ s^−1^, approximately three times higher than ZnPc (0.31 × 10^22^ cm^−3^ s^−1^) under similar excitation, demonstrating its superior continuous carrier supply capability [19,20]. Beyond its exceptional HCGR, TiOPc possesses broad near-infrared absorption, tunable polymorphism via molecular stacking, and a planar π-conjugated structure that enhances electronic stability [21,22]. These properties also facilitate analyte adsorption, strengthen excitonic interactions, and enable precise tuning of interfacial CT for reproducible SERS [23,24,25,26]. By integrating TiOPc with atomically thin MoS_2_, we constructed a heterojunction that synergises TiOPc’s HCGR-driven carrier generation with MoS_2_’s efficient transport [27]. This HCGR/ECT coupling establishes a dynamic enhancement mechanism that fundamentally surpasses static band alignment constraints. The high carrier flux enables ECT across the π-conjugated TiOPc/MoS_2_ interface, promoting exciton dissociation, spatially separated interlayer excitons with extended lifetimes, and enhanced dipole moments [10,22,28,29]. Critically, this ECT process facilitates photoinduced charge exchange with analytes, modulates resonance through energy-level realignment, and reshapes the local electronic environment, thereby amplifying CM-dominated SERS (Figure S10). Although semiconductor SERS substrates (EF: 10^3^–10^6^) are behind noble metals [2,10,22,30,31], the van der Waals TiOPc/MoS_2_ heterostructure offers a promising pathway by addressing the core limitations of carrier density and transfer dynamics. A mechanistic understanding of HCGR-driven ECT is thus pivotal for designing next-generation SERS sensing platforms.

This study introduces a TiOPc/MoS_2_ hybrid platform that overcomes the kinetic limitations of conventional semiconductor SERS substrates through HCGR-enabled ECT. We establish HCGR as a kinetic design parameter that transcends static band engineering, enabling enhancement factors of 1.56 × 10^7^ (MB) and 1.61 × 10^7^ (R6G) with detection limits of 10^−14^ M and 10^−13^ M, respectively. The platform demonstrates exceptional reproducibility (RSD = 7.2%) and 60-day stability. Furthermore, ECT accelerates MB photodegradation under UV light (complete decomposition in 80 min) while preserving SERS activity. Environmental monitoring capabilities are evidenced by detecting Hg^2+^ (LOD: 10^−11^ M) with minimal interference from other metal ions and malachite green in tap/lake water (LODs: 10^−12^ M/10^−10^ M). This work establishes HCGR-driven ECT as a universal design principle for high-performance semiconductor SERS sensors, with transformative potential for real-time industrial wastewater monitoring and pollutant degradation tracking.

## 2. Results and Discussion

### 2.1. Characterization of TiOPc/MoS_2_ Nanocomposite

#### 2.1.1. Structural and Morphological Characterization

**Scheme 1:**
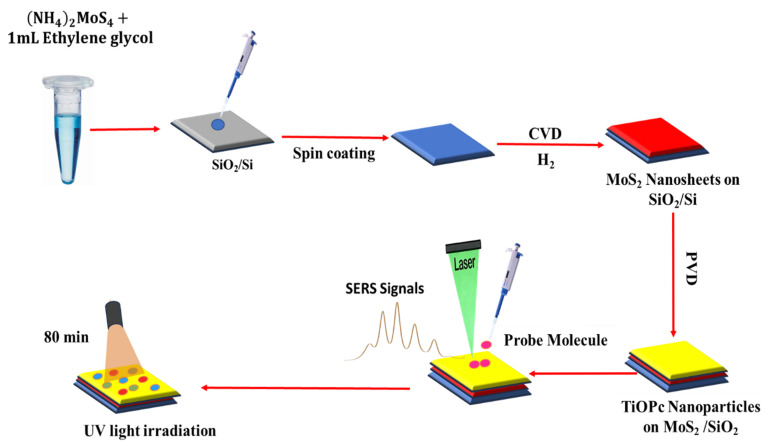
Schematic illustration of TiOPc/MoS_2_ heterostructure and SRES experiments.

**Figure 1:**
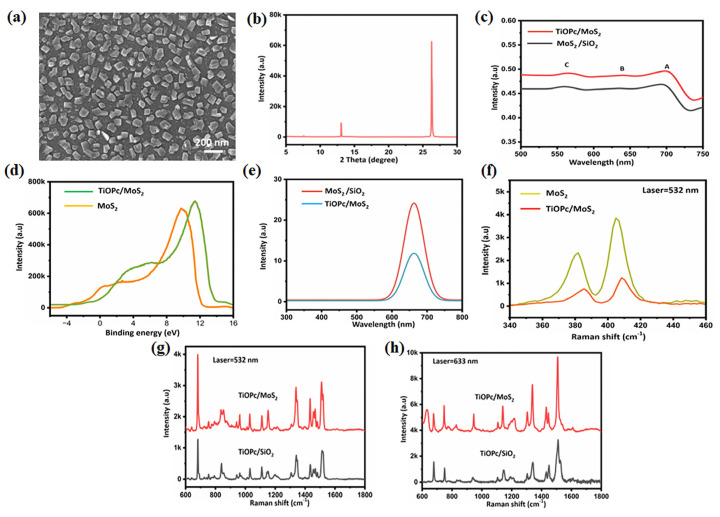
Structural, optical, and interfacial characterization of the TiOPc/MoS_2_ heterostructure: (**a**) SEM image, (**b**) XRD pattern, (**c**) UV–Vis absorption spectra, (**d**) UPS spectra, (**e**) PL spectra, (**f**) Raman spectra of MoS_2_ and TiOPc/MoS_2_ in the range 360–440 cm^−1^, and Raman spectra of TiOPc/SiO_2_ and TiOPc/MoS_2_ under (**g**) 532 nm and (**h**) 633 nm excitation.

Scheme 1 illustrates the preparation of the TiOPc/MoS_2_ heterostructure and its application in SERS and photodegradation experiments. The growth performance of TiOPc on surface of MoS_2_ was examined by SEM at different deposition times while keeping the temperature and substrate distance constant. As shown in Figure S1a–c, deposition for 4 min produces small, sparsely distributed TiOPc particles with low surface coverage. Increasing the deposition time to 10 min results in larger and closely packed plate-like crystallites, whereas prolonged deposition for 13 min leads to excessive growth, forming large overlapping TiOPc plates and a compact surface. The SEM image of the TiOPc/MoS_2_ heterostructure prepared at 7 min, shown in Figure 1a, reveals a relatively uniform distribution of TiOPc nanoparticles on the MoS_2_ surface. The corresponding particle-size distribution is provided in Figure S2. These nanoparticles exhibit crystalline and faceted morphologies with a non-uniform size distribution. The partial exposure of the underlying MoS_2_ suggests an island-like Volmer–Weber growth mode, likely arising from weak van der Waals interactions between TiOPc and MoS_2_, together with stronger intermolecular interactions within TiOPc. Surface defects and step edges on MoS_2_ may also act as nucleation sites, promoting nanoparticle dispersion [25]. This island-like TiOPc morphology is beneficial for SERS because it increases the interfacial contact area with MoS_2_, supports interfacial charge transfer, and generates localized hot spots. The intimate TiOPc/MoS_2_ contact shortens the charge-transfer distance and strengthens electronic coupling, providing a structural basis for efficient charge transfer and separation. In addition, the HCGR-driven molecular ordering of TiOPc may improve carrier mobility and enhance carrier flux across the heterostructure. However, the structural non-uniformity associated with island-like growth may affect the long-term reliability of the sensor, particularly for large-scale industrial applications. Irregular TiOPc coverage can cause non-uniform hot-spot distribution, spatial variation in charge-transfer efficiency, and restricted lateral charge transport across the substrate. These effects may lead to signal fluctuation, reduced spot-to-spot reproducibility, and variation in sensing performance during extended use or large-area fabrication. Therefore, although the island-like morphology enhances local SERS activity, further optimization of film uniformity is needed to improve sensor reliability, scalability, and batch-to-batch consistency. Future studies may explore layer-by-layer assembly, Langmuir–Blodgett deposition, organic molecular beam deposition, solvent-engineered spin coating, dip coating, drop-casting, or seed-layer-assisted growth to improve film continuity while maintaining SERS enhancement. The successful formation of the TiOPc/MoS_2_ heterostructure is further confirmed by EDS elemental mapping, as elaborated in Figure S3. The wettability of different SERS substrates was also evaluated using water contact-angle measurements, as discussed in Figure S4.

#### 2.1.2. Crystallographic and Interfacial Properties

The XRD pattern of the TiOPc/MoS_2_ heterostructure (Figure 1b) displays three different reflections at 2θ angles of 7.6°, 13.05°, and 26.3°. Interplanar spacings (d) were calculated utilizing Bragg’s rule (nλ = 2d sin θ, with λ = 1.5418 Å for Cu Kα radiation). The 7.6° peak correlates to a d-spacing of around 11.6 Å, corresponding to the long-range molecular stacking of TiOPc in the γ-phase layered configuration, in agreement with other studies on vacuum-deposited TiOPc thin films [32,33]. The 13.05° peak produces a d-spacing of approximately 6.8 Å, likely linked to higher-order reflections, resulting from in-plane molecular organization inside the phthalocyanine lattice [22]. The dominant peak at 26.3° correlates with a d-spacing of around 3.4 Å, aligning with the typical π–π stacking distance of phthalocyanine macrocycles. This pronounced reflection, frequently noted in crystalline TiOPc films, indicates a preferred alignment of columnar stacks perpendicular to the substrate [22,33]. The comparative peak intensities elucidate the crystallization behavior of TiOPc. The 26.3° reflection has an intensity of around 62,479 counts, markedly surpassing the 7.6° (~760 counts) and 13.05° (~9169 counts) maxima. The intensity distribution validates the predominance of π–π stacking in the crystallographic arrangement of TiOPc nanoparticles, signifying highly organized domains with a vertical stacking preference, characteristic of phthalocyanine molecules formed during physical vapor deposition. The diminished reflections at lower angles arise from fewer densely arranged lattice surfaces, which scatter X-rays with reduced efficacy. The XRD pattern conspicuously lacks discernible diffraction peaks from MoS_2_. The lack of the MoS_2_ (002) peak at about 14° is due to the ultrathin tri layer configuration of MoS_2_, which offers inadequate scattering volume to generate observable diffraction intensity in standard θ–2θ XRD. This behaviour has been noted in other organic/2D heterostructures, where intense diffraction from crystalline organic overlayers masks the faint signals from atomically thin MoS_2_ [25,34,35].

The UV–Vis absorption spectra for different substrates, with SiO_2_ serving as a reference, are illustrated in Figure 1c and Figure S5a. The bare SiO_2_ substrate (Figure S5a) exhibits no notable absorption features, thereby confirming its optical transparency. The TiOPc/SiO_2_ sample (Figure S5a) displays two distinct absorption bands at 420.5 nm and 730.5 nm, which correspond to the B (Soret-like) and Q bands of TiOPc, respectively, consistent with previously reported values [36]. In the case of MoS_2_/SiO_2_ (Figure 1c), two distinct absorption peaks are identified at 633.72 nm (B exciton) and 694.72 nm (A exciton), aligning with the intrinsic excitonic transitions of MoS_2_ due to spin-orbit splitting of the valence band at the K point [2,25]. The deposition of TiOPc on MoS_2_ results in a significant alteration of the absorption profile. The MoS_2_ A and B exciton peaks shifted to longer wavelengths, at 700.5 nm and 640.5 nm, respectively. Additionally, the TiOPc Q band at 730.5 nm overlaps with the MoS_2_ A exciton, as illustrated in Figure 1d and Figure S5a. The observed overlap indicates electronic coupling and interfacial interaction between TiOPc molecular states and MoS_2_ excitonic transitions, which modifies the exciton resonance energies [2]. The red shift of MoS_2_ excitons in the TiOPc/MoS_2_ heterostructure, relative to MoS_2_/SiO_2_, is ascribed to enhanced dielectric screening and CT mechanisms at the organic/2D interface [25,37]. The spectral overlap between the TiOPc Q band and the MoS_2_ A exciton provides additional evidence for the establishment of a coupled absorption system. Furthermore, the TiOPc/MoS_2_ composite exhibits a stronger absorption than MoS_2_/SiO_2_ in the range of 500–750 nm and displays distinct features associated with MoS_2_. This establishes that TiOPc serves as the primary light-harvesting and, under HCGR conditions, is capable of generating and transmitting a substantial carrier flux to the interface. The peaks reshaping in the composite indicate interfacial coupling, aligning with ECT in TiOPc/MoS_2_. The findings demonstrate robust interfacial coupling, which amplifies exciton-molecule interaction and promotes ECT inside the hybrid structure, which is a vital effect for enhancing the SERS activity. Meanwhile, according to the Tauc plot results of the materials, the obtained band gap (Figure S5b) of TiOPc/SiO_2_, MoS_2_/SiO_2_, and TiOPc/MoS_2_ is 1.75, 1.64, and 1.54 eV, respectively.

Ultraviolet photoelectron spectroscopy investigation of MoS_2_ and the TiOPc/MoS_2_ heterostructure demonstrated notable alterations in the valence band structure, as shown in Figure 1d. The valence band maximum increased by roughly 1.1 eV, rising from −4.05 eV in pristine MoS_2_ to −2.95 eV following TiOPc deposition. The observed alterations indicate CT and band bending at the interface, with TiOPc functioning as an electron acceptor and facilitating p-type doping in MoS_2_ [25,38]. This interaction results in the formation of an interface dipole, hence decreasing the effective work function. The enhanced spectrum intensity is probably due to the overlap of TiOPc molecular orbitals with MoS_2_ valence levels, hence confirming hybridization and a high density of states. The increased density of states at the Fermi level in the TiOPc/MoS_2_ heterostructure facilitates CT between the molecule and substrate, hence reinforcing interfacial coupling [6]. Furthermore, the TiOPc overlayer may mitigate sulphur vacancies associated with defect states, resulting in a more defined band edge [38,39]. The results demonstrate that TiOPc/MoS_2_ establishes a type-II heterojunction with advantageous band alignment for charge separation, defect mitigation, and interfacial stability. These features allow this heterostructure to be advantageous for SERS sensing applications. Meanwhile, XPS was conducted to analyze the chemical states and interfacial electronic interactions of MoS_2_ and TiOPc/MoS_2_**,** with detailed spectra discussed in Figure S6.

The photoluminescence (PL) spectra demonstrate unique emission characteristics for the individual components and their stacked heterostructures. Bare SiO_2_ (Figure S7) displays a broad band centered at around 450.10 nm, indicative of defect-related radiative centers, including oxygen-deficiency sites and non-bridging oxygen hole centers [40]. Following the deposition of TiOPc on SiO_2_, the defect emission is significantly diminished, and a new band appears at around 524.21 nm, attributed to aggregate-induced emission from TiOPc in the solid state [41]. This alteration aligns with the optical behavior depending on aggregation and polymorphism frequently reported in TiOPc molecular thin films [42]. Figure 1e illustrates that tri layer MoS_2_ has a significant excitonic PL peak at approximately 664 nm, which corresponds to the direct A-exciton transition. Following the deposition of TiOPc, the peak location shifts slightly to approximately 663 nm, while the emission intensity significantly diminishes, signifying strong exciton quenching inside the TiOPc/MoS_2_ heterostructure. This strong quenching (in Figure 1e) coincides with strong UV–Vis absorption (Figure 1c), suggesting improved light harvesting and, hence, an increased photocarrier generation rate under the same excitation circumstances. The strong quenching of PL, despite heightened carrier production, suggests that photoexcited carriers are effectively redirected from radiative recombination towards interfacial relaxation routes. This phenomenon aligns with ECT, namely interfacial electron or hole transfer happening in a short duration that can quench exciton recombination in MoS_2_. In a type-II aligned TiOPc/MoS_2_ heterostructure, the interfacial energetic driving force facilitates a directional extraction pathway that swiftly depletes photogenerated carriers from MoS_2_ into TiOPc, thereby inhibiting A-exciton emission [22,25]. While steady-state PL and absorption spectra alone do not measure transfer kinetics, the conjunction of widened absorbance (indicating high charge generation rates) and strong photoluminescence quenching around it provides compelling spectroscopic evidence for effective and ECT separation. These results demonstrate significant electronic coupling at the TiOPc/MoS_2_ interface and elucidate how interfacial interactions among TiOPc, MoS_2_, and the SiO_2_ substrate influence the optical response of the layered structures.

#### 2.1.3. Raman Properties of MoS_2_ and TiOPc/MoS_2_ Composites

The analysis of TiOPc/MoS_2_ composites using Raman spectroscopy elucidates the influence of TiOPc nanostructures on 2D MoS_2_ films. Figure 1f shows the Raman spectra of TiOPc/MoS_2_ composites and pristine 2D MoS_2_. The spectra were taken with a 532 nm excitation laser in the 360–460 cm^−1^ range. The selected wavelength exhibits strong resonance with the first-order Raman-active modes of MoS_2_, thereby enhancing sensitivity to phonon features and facilitating accurate detection of structural changes induced by TiOPc deposition [6,25,37]. The MoS_2_ spectrum displays E_2g_ and A_1g_ peaks at 381.8 cm^−1^ and 404.9 cm^−1^, respectively, with a frequency difference of 23 cm^−1^, suggesting trilayer MoS_2_ [25]. In the TiOPc/MoS_2_ composites, the peaks shift to 385.4 cm^−1^ (E_2g_) and 408.5 cm^−1^ (A_1g_), indicating interactions between the materials without notable lattice deformation, as the frequency difference remains unchanged. The stability of TiOPc/MoS_2_ composites is mainly due to non-covalent bonding and van der Waals forces, which are enhanced by π-π stacking interactions between the conjugated π-electron systems of MoS_2_ and TiOPc molecules. Although there are differences in lattice constants between TiOPc and MoS_2_, the materials accommodate one another with minimal lattice deformation, thereby minimizing mechanical stress resulting from lattice mismatch [22,25]. Concurrently, the overall peak intensity is markedly diminished, and the bands exhibit broadening, attributable to electronic interactions at the interface, including CT and phonon damping effects induced by the TiOPc overlayer [6,25].

The influence of laser excitation wavelength on the Raman spectra of TiOPc is well illustrated in Figure 1g, h. Upon excitation at 532 nm, the most pronounced Raman signals are observed at approximately 680 and 682 cm^−1^ for TiOPc/MoS_2_ and TiOPc/SiO_2_, respectively. Under 633 nm excitation, the predominant characteristic moves to about 1508 cm^−1^ for TiOPc/MoS_2_ and 1510 cm^−1^ for TiOPc/SiO_2_. This disparity originates from resonance that depends on wavelength [43]. The 633 nm laser, positioned near the Q-band absorption of TiOPc (Figure S4a), preferentially enhances Raman signals by amplifying Cα-Nα-Cα scissoring and Cα-Nβ-Cα stretching asymmetrical vibrations that are closely coupled to π-π* electronic transitions of the macrocycle. In contrast, 532 nm excitation investigates an alternative vibronic resonance, particularly amplifying Cα-Nα-Cα wiggling and C-H out-of-plane vibrations. Laser stimulation not only enhances the Raman intensity of several Raman modes at distinct places but also induces shifts in the wavenumbers of these modes. The relative strength of these bands is further increased and their Raman modes altered when TiOPc is deposited on MoS_2_, indicating chemical enhancement via ECT, which promotes greater electron–phonon interaction at the hybrid interface. Mode assignments [23,24] and band changes in Raman peaks (Table S1) offer additional understanding of the vibrational behavior of TiOPc under these circumstances.

### 2.2. SERS Properties of TiOPc/MoS_2_ Nanocomposite

R6G and MB were used as probe molecules to evaluate the SERS performance of the TiOPc/MoS_2_ substrate due to their complementary optical properties. R6G exhibits a strong Raman cross-section and pronounced resonance under visible excitation (489–524 nm, Figure S8a), producing intense and well-defined vibrational peaks suitable for estimating enhancement factors under 532 nm excitation. In contrast, MB absorbs in the red/NIR region (~660 nm, Figure S8b), making it appropriate for assessing SERS activity under 633 nm irradiation. Beyond their optical differences, the dyes also vary in molecular structure and adsorption behavior: R6G primarily interacts through π–π stacking, while MB contains nitrogen and sulphur heteroatoms capable of coordinating with semiconductor surface sites [44]. The utilization of both dyes enables evaluation of resonance effects, adsorption chemistry, and substrate reproducibility, providing a comprehensive assessment of the SERS substrate’s performance and applicability.

**Figure 2:**
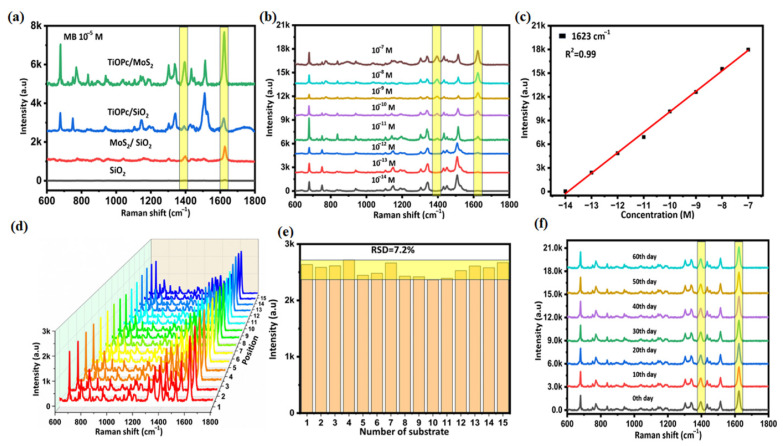
(**a**) Raman spectra of MB (10^−5^ M) on different substrates. (**b**) SERS spectra of MB on TiOPc/MoS_2_ at concentrations ranging from 10^−14^ to 10^−7^ M. (**c**) Correlation between MB concentration and SERS intensity at 1623 cm^−1^. (**d**) SERS reproducibility of MB (10^−5^ M) on TiOPc/MoS_2_ measured at fifteen random locations. (**e**) Repeatability of the 1623 cm^−1^ peak for MB. (**f**) Stability of the TiOPc/MoS_2_ SERS substrate over 60 days.

Figure 2a presents a comparison of the SERS spectra of MB (10^−5^ M) across different substrates. The calculated enhancement factor of TiOPc/SiO_2_, MoS_2_/SiO_2_, and TiOPc/MoS_2_ is 2.5 × 10^3^, 3.8 × 10^3^, and 1.56 × 10^7^, respectively. The TiOPc/MoS_2_ hybrid exhibits the most pronounced Raman response due to synergetic effect of organic and inorganic, specifically at 1437 and 1625 cm^−1^, which are associated with the C–N stretching and aromatic C–C ring stretching modes of MB [25]. A comparison of the EF and LOD of TiOPc/MoS_2_ with previously reported SERS substrates is presented in Table S2, demonstrating that TiOPc/MoS_2_ is the best among the reported SERS substrates. This superior SERS performance of the TiOPc/MoS_2_ heterostructure originates from the exceptionally HCGR of TiOPc, which drives ECT across the interface, enabling efficient charge separation and rapid exciton dissipation; consequently, chemical enhancement dominates the SERS response, while local electromagnetic field enhancement plays a secondary role. Figure 2b presents the concentration-dependent SERS spectra of MB ranging from 10^−7^ to 10^−14^ M on TiOPc/MoS_2_. With a decrease in concentration, Raman intensity consistently declines. At higher concentrations, the peaks of MB are predominant; conversely, at lower concentrations (≤10^−11^ M), the intrinsic Raman modes of TiOPc gain prominence as a result of diminished CT coupling between MB and the substrate. The calibration plot in Figure 2c demonstrates a linear correlation between Raman intensity and MB concentration (R^2^ ≈ 0.99), thereby confirming the reliability of quantitative sensing capabilities and the consistency of CT-mediated enhancement. Figure 2e,d demonstrate the spatial uniformity and reproducibility of the SERS response. 3D Raman mapping demonstrates some variation in SERS spectral intensities throughout measurement points, exhibiting a relative standard deviation (RSD) of 7.2%. The little bit of variation in SERS spectral intensities arises from the distribution of TiOPc nanoparticles on MoS_2_, which ensures interfacial electronic coupling. The long-term stability of the TiOPc/MoS_2_ substrate is assessed in Figure 2f. Following 60 days of ambient storage, the Raman spectra show only about a 10% decrease in intensity with negligible spectral distortion, demonstrating the excellent chemical and environmental stability of the TiOPc/MoS_2_ hybrid substrate. This enhanced stability is primarily attributed to the combined effects of the tri-layer MoS_2_ structure [45] and the chemically inert TiOPc [46] overlayer under ambient conditions. Although surface oxidation is a known limitation of monolayer MoS_2_, bilayer and multilayer MoS_2_ are less susceptible to ambient oxidation due to an indirect band gap, thereby reducing degradation during prolonged exposure and up to two years [45]. In addition, the chemically stable TiOPc overlayer forms a stable van der Waals interface with MoS_2_ and shields the surface from water molecules and OH radicals, further suppressing oxidation. Moreover, TiOPc passivate defect-related trap states in MoS_2_ through interfacial charge transfer [39]. This is supported by the Raman spectra in Figure 1f, where no obvious structural distortion was observed in the TiOPc/MoS_2_ hybrid, which is associated with defect-related states [37]. Therefore, the long-term stability of TiOPc/MoS_2_ can be attributed to physical surface protection and electronic passivation, which together maintain its charge-transfer-assisted SERS activity under ambient conditions. The SERS performance of the TiOPc/MoS_2_ hybrid substrate was further evaluated using R6G as a probe molecule, as detailed in Figure S9. These findings demonstrate that the TiOPc/MoS_2_ hybrid substrate exhibits enhanced SERS activity, reproducibility, and long-term stability, rendering it appropriate for ultrasensitive and dependable molecular detection.

### 2.3. SERS Enhancement Mechanisms of TiOPc/MoS_2_ SERS Substrate

The combined UV–Vis, PL, and XPS results collectively demonstrate enhanced photoexcitation, improved carrier extraction with suppressed radiative recombination, and interfacial charge redistribution/chemical-state modulation. These effects synergistically increase the HCGR and facilitate internal-field-assisted efficient charge transfer (ECT) into active sites.

**Figure 3:**
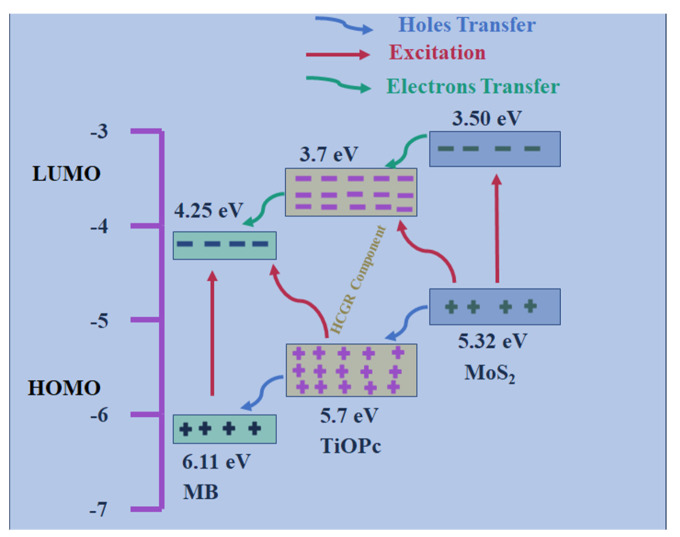
Schematic of energy levels alignment of MB and TiOPc/MoS_2_ heterostructure for SERS enhancement.

The charge transfer (CT) behavior of the TiOPc/MoS_2_ heterostructure, supported by density functional theory (DFT) calculations detailed in the Supporting Information (Figure S10). Figure 3 depicts the interfacial charge-transport pathway, employing MB as a probe molecule to reveal the dynamic enhancement mechanism, which was derived from prior studies [25,37,47]. The system functions within a donor–mediator–acceptor framework: MoS_2_ acts as the photoexcited semiconductor donor, TiOPc serves as an interfacial mediator generating a high carrier flux under illumination, and MB operates as the ultimate charge-accepting molecular site. Notably, the extraordinary enhancement (≈10^7^) and ultralow detection limit (≈10^−14^ M) cannot be accounted for static energy-level alignment alone, necessitating a kinetic explanation. Upon illumination, TiOPc maintains a nonequilibrium interfacial carrier population, effectively providing HCGR and functioning as an electronic bridge that enables efficient charge transfer to adsorbed MB while concurrently suppressing recombination and back-transfer. This ECT-driven, high-flux interfacial transport repeatedly generates transient CT states localized on MB, which enhances molecular polarizability and vibronic coupling, thereby amplifying the Raman scattering cross-section. This observation aligns with chemical enhancement models that prioritize kinetically sustained CT processes over equilibrium orbital alignment [10,28].

The electromagnetic enhancement in the TiOPc/MoS_2_ SERS substrate is relatively weak because both MoS_2_ and TiOPc are semiconducting materials with limited plasmonic resonance compared with noble-metal-based substrates. Therefore, the SERS performance is mainly governed by the chemical mechanism rather than the electromagnetic mechanism. As shown by the COMSOL simulation in Figure S11 (SI), the electromagnetic enhancement factor was estimated using the formula EF_EM_ ≈ |E/E_0_|^4^ and was found to be approximately 2.1 × 10^4^. This value is much lower than the experimentally measured enhancement factor of about 10^7^. This difference indicates that the strong SERS activity of the TiOPc/MoS_2_ substrate mainly arises from efficient interfacial charge transfer and advantageous of energy-level coupling among TiOPc, MoS_2_, and the probe molecules. Electromagnetic hot spots may still contribute to the overall enhancement, but their role is secondary and supportive in this semiconductor-based SERS system.

### 2.4. Photodegradation Activity of TiOPc/MoS_2_ SERS Substrate

**Figure 4:**
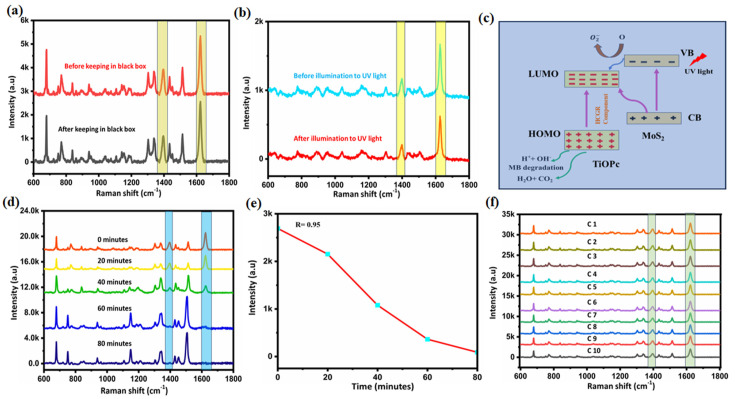
Photocatalytic degradation of MB using the TiOPc/MoS_2_ hybrid substrate. (**a**) SERS spectra recorded before and after keeping the MB/TiOPc/MoS_2_ system in the dark for 80 min, (**b**) SERS spectra of MB before and after UV irradiation on MoS_2_. (**c**) Proposed photocatalytic degradation mechanism of MB over the TiOPc/MoS_2_ hybrid substrate. (**d**) Time-dependent Raman spectra of MB in the presence of TiOPc/MoS_2_ under UV irradiation from 0 to 80 min. (**e**) Decay of MB SERS intensity with irradiation time, showing degradation behavior with R^2^ = 0.95 at 1623 cm^−1^. (**f**) SERS spectra recorded over ten repeated photocatalytic degradation cycles, confirming the stability and reusability of the TiOPc/MoS_2_ substrate.

Before UV irradiation, the MB/TiOPc/MoS_2_ system was kept in complete darkness for 80 min by placing it in a black box and covering it with a black sheet to establish adsorption-desorption equilibrium. The Raman spectra recorded before and after dark treatment showed no significant decrease in MB intensity, confirming that MB adsorption on the catalyst surface in the dark was negligible, as shown in Figure 4a. As shown in Figure 4b, after 80 min of UV irradiation on paritine MoS_2_, only a small decrease in MB Raman intensity is observed, and the characteristic MB peaks remain clearly visible. This result shows that UV light alone has a limited effect on MB degradation. In contrast, the much stronger decrease in MB signal in the presence of TiOPc/MoS_2_ confirms that the hybrid substrate is mainly responsible for the enhanced photocatalytic degradation of MB. Figure 4c schematically depicts the photocatalytic degradation mechanism of the TiOPc/MoS_2_ nanocomposite, functioning as a Type-II CT heterojunction. Both TiOPc nanoparticles and MoS_2_ are photoexcited under UV light irradiation. In TiOPc, electrons are elevated from the highest occupied molecular orbital (HOMO) to the lowest unoccupied molecular orbital (LUMO), but in MoS_2_, electrons migrate from the valence band (VB) to the conduction band (CB). The elevated carrier generation rate of TiOPc results in a substantial population of photogenerated charge carriers under continuous illumination. The type-II heterojunction enables effective ECT at the interface, with photoexcited electrons migrating from MoS_2_ to the LUMO of TiOPc, while holes are retained in the VB of MoS_2_ or the HOMO of TiOPc. The interfacial CT diminishes electron–hole recombination and guarantees prolonged carrier lifetimes, hence improving photocatalytic efficiency. The electrons amassed in the LUMO of TiOPc enable the reduction of molecular oxygen, resulting in the formation of superoxide radicals (O_2_^−^), whereas the holes in TiOPc or MoS_2_ enhance the oxidation of water or hydroxide ions, producing hydroxyl radicals (OH^−^). The ongoing production of reactive oxygen species, driven by HCGR and ECT, is essential for the breakdown of methylene blue, resulting in its mineralization into CO_2_ and H_2_O. The photocatalytic activity of the TiOPc/MoS_2_ heterostructure is facilitated by the Type-II CT mechanism, wherein effective charge separation, supported by high carrier generation rate and ECT, is essential for augmenting the generation of reactive oxygen species, crucial for the photocatalytic degradation process [48]. Figure 4d displays the Raman spectra obtained at different illumination durations (0, 20, 40, 60, and 80 min). The characteristic peaks of MB, indicated in the shaded regions, progressively decrease with increasing irradiation time, demonstrating a steady decline from approximately 2700 a.u. to nearly zero over a duration of 80 min. The comparison of photodegradation activity of TiOPc/MoS_2_ SERS substrate with previously reported SERS substrates is presented in Table S3. The observed decline suggests a steady decrease in MB concentration with increasing irradiation time, exhibiting behavior consistent with first-order kinetics, with an R^2^ value of 0.95 at 1623 cm^−1^, characteristic of photocatalytic degradation processes [49,50], as depicted in Figure 4e. This phenomenon is propelled by effective electron–hole separation at the TiOPc/MoS_2_ interface, enabled by the high carrier generation rate in TiOPc, which drives ECT between TiOPc and MoS_2_. This process diminishes recombination, extends charge lifetimes, and enhances the formation of reactive species, hence accelerating the degradation of methylene blue and elucidating the rapid reduction in Raman signals with prolonged illumination [30]. To evaluate the photocatalytic self-cleaning stability and reusability of the TiOPc/MoS_2_ substrate, cyclic MB degradation experiments were performed for ten consecutive cycles under UV irradiation. Each cycle was conducted for 80 min under the same experimental conditions, corresponding to a total irradiation time of 800 min. After each cycle, the substrate was carefully washed to remove residual MB and loosely adsorbed degradation species before being reused for the next cycle. As shown in Figure 4f, the SERS spectra recorded after each cycle show a gradual decrease in the characteristic MB peak intensity from cycle 1 to cycle 10, confirming the repeated photocatalytic self-cleaning activity of the TiOPc/MoS_2_ heterostructure. The corresponding intensity variation plotted as a function of total irradiation time, shown in Figure S12, further shows a gradual decline, with the Raman intensity decreasing by approximately 27% after the 10th cycle compared with the first cycle. This moderate decrease may be attributed to partial surface fouling or slight changes at the active TiOPc/MoS_2_ interface during repeated UV exposure. Importantly, the substrate still retained a clear Raman response after ten cycles, indicating that the TiOPc/MoS_2_ interface remained largely stable without severe degradation or delamination. These results demonstrate that the TiOPc/MoS_2_ heterostructure possesses good photocatalytic self-cleaning capability and reasonable reusability, supporting its potential for repeated sensing and regeneration applications. The integration of SERS detection with catalytic degradation on a single platform has been demonstrated as a promising strategy for organic pollutant removal [51].

### 2.5. Application of TiOPc/MoS_2_ SERS Substrate for the Detection of MG and Hg^2+^ Ions

The SERS performance of the TiOPc/MoS_2_ substrate was evaluated using R6G and MB under 532 nm and 633 nm excitation, respectively. To demonstrate practical applicability, MG and 4MPy–Hg^2+^ (with 4MPy as the Raman reporter) were selected to represent both excitation conditions. MG exhibits strong absorption in the visible region (~620–650 nm) [52], enabling resonant Raman enhancement under 633 nm excitation, while 4MPy absorbs in the UV region (<320 nm) [53], so its 532 nm-excited SERS signal mainly originates from chemical enhancement via strong surface bonding and charge-transfer interactions. Hg^2+^ and MG are highly toxic environmental pollutants with persistence and bioaccumulation risks [54,55]. Hg^2+^ disrupts metabolic and neurological functions and can convert into toxic methylmercury [56,57]. MG, which is used in textiles and aquaculture, is mutagenic and carcinogenic [55,58,59,60]. Therefore, SERS offers a sensitive and selective method for detecting such hazardous contaminants in the environment [2,6,61,62].

**Figure 5:**
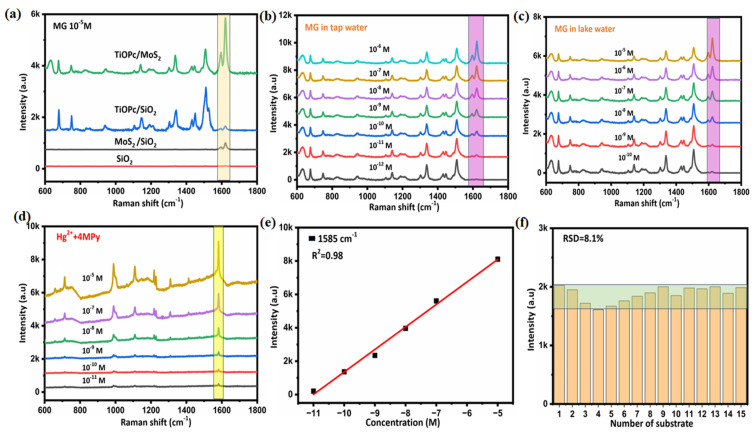
(**a**) SERS spectra of MG (10^−5^ M) on different substrates. (**b**) SERS spectra of MG on TiOPc/MoS_2_ at concentrations ranging from 10^−12^ to 10^−7^ M. (**c**) SERS detection of MG in lake water at concentration ranging from 10^−10^ to 10^−5^ M on TiOPc/MoS_2_. (**d**) SERS spectra of Hg^2+^ on TiOPc/MoS_2_ at concentration ranging from 10^−11^ to 10^−5^ M. (**e**) Correlation between Hg^2+^ concentration and SERS intensity at 1585 cm^−1^. (**f**) Repeatability of the 1585 cm^−1^ peak for Hg^2+^ detection on different substrates.

Figure 5a shows the SERS spectra of MG at 10^−5^ M on different substrates, including SiO_2_, MoS_2_/SiO_2_, and TiOPc/MoS_2_. The characteristic peaks at 1622 and 1595 cm^−1^ are assigned to aromatic C–C stretching and N–phenyl stretching vibrations, respectively [61,63]. Among these substrates, TiOPc/MoS_2_ exhibits the strongest SERS response, which is attributed to the synergistic interaction between organic TiOPc and inorganic MoS_2_. This interaction enhances efficient interfacial charge transfer and local electromagnetic fields, thereby improving the Raman SERS signals of MG. Figure 5b presents the SERS spectra of MG on TiOPc/MoS_2_ across various concentrations (10^−7^–10^−12^ M), illustrating a progressive reduction in intensity with dilution, while distinctly recognizable peaks persist down to 10^−11^ M. The comparison of the limit of detection (LOD) of TiOPc/MoS_2_ with previously reported SERS substrates is presented in Table S4, indicating that TiOPc/MoS_2_ is the most effective among the reported SERS substrates. Figure S13a illustrates the calibration plot of Raman intensity at 1622 cm^−1^ against MG concentration, revealing a linear relationship with a correlation coefficient of R^2^ ≈ 0.99. This observation suggests a reliable quantitative detection capability and consistent surface adsorption behavior. The SERS spectra of MG in lake water in the range 10^−5^ to 10^−10^ M, as shown in Figure 5c, collected from the lake of Shandong Normal University, exhibit slightly shifted peaks at 1620 and 1593 cm^−1^. The observed minor redshifts are ascribed to interactions between MG molecules and ionic or organic constituents in natural water, which can modify the local dielectric environment and the molecular adsorption configuration on the TiOPc/MoS_2_ surface. The plot of intensity versus concentration in lake water displays a linear trend with R^2^ ≈ 0.99, as shown in Figure S13b, thereby affirming the substrate’s significant sensitivity and practical utility for environmental monitoring. Figure S13c illustrates that uniformity mapping demonstrates consistent spectral profiles across various positions, exhibiting only minor intensity variations of the MG in lake water. The reproducibility test conducted on fifteen independent substrates results in a relative standard deviation (RSD) of 9.5%, as illustrated in Figure S13d, indicating high signal reproducibility and structural consistency of the MG in lake water on the TiOPc/MoS_2_ SERS substrate. Meanwhile, Mg recovery was higher in deionized water than in lake water due to the absence of matrix components and minimal signal interference. The lower recoveries observed in lake water are attributed to dissolved ionic species and naturally occurring matrix components that can affect the analytical response. All Mg recovery values in deionized water and lake water are summarized in Table S5.

In SERS-based metal ion detection, 4-mercaptopyridine (4MPy) is used as a Raman reporter molecule, producing strong and chemically responsive Raman signals that reflect interactions between the substrate and target ions [64]. The TiOPc/MoS_2_ was initially immersed in a 4MPy solution for 30 min and then allowed to dry under ambient condition before being utilized for SERS detection of Hg^2+^ ions. The 4MPy molecule binds to the TiOPc/MoS_2_ surface through its thiol group [6], and its pyridine nitrogen exhibits selective binding to Hg^2+^ ions [64,65]. Figure S14a illustrates the SERS spectra of TiOPc/MoS_2_ prior to and after to treatment with 4-MPy. The unblemished TiOPc/MoS_2_ composite displays Raman signals mostly attributed to the vibrational modes of MoS_2_ and the molecular vibrations of TiOPc. Following functionalization with 4-MPy, several discrete peaks emerge at 722, 988, 1044, 1199, 1399, and 1619 cm^−1^. These pronounced and refined peaks indicate robust adsorption of 4-MPy molecules and effective CT interaction between 4-MPy and the TiOPc/MoS_2_ substrate, resulting in substantial SERS enhancement. The introduction of Hg^2+^ ions (Figure 5d) results in a significant alteration of the SERS profile. The principal peaks transition to 713, 988, 1217, 1309, 1413, and 1585 cm^−1^, accompanied by significant intensity and shape fluctuations, which all are in good agreement with the reported literature [66]. The redshift of the ring-breathing mode (from 722 to 713 cm^−1^) signifies cooperation between Hg^2+^ ions and the nitrogen atom of the pyridine ring, and significant intense band at 1585 cm^−1^ emerges, indicative of an intensified C=C/C–N stretching vibration [64,67]. This intensely identified characteristic is not present in the original SERS spectrum of 4-MPy on TiOPc/MoS_2_ (Figure S14a), validating the creation of a Hg–N coordination complex, which alters the molecular electronic environment and amplifies CT interaction between 4-MPy and the TiOPc/MoS_2_ interface. Figure 5d illustrates the appearance of the 1585 cm^−1^ peak following exposure to Hg^2+^, thereby confirming the interaction between Hg^2+^ and 4MPy. The peak intensity rises with increasing Hg^2+^ concentration from 10^−11^ to 10^−5^ M, demonstrating the sensitivity of the 4MPy-TiOPc/MoS_2_ hybrid substrate. The reported LOD of 10^−11^ M refers to the lowest detectable concentration of Hg^2+^ ions in the bulk solution under the applied incubation and rinsing protocol. It does not represent the final surface concentration of Hg^2+^ retained on the substrate after washing. This LOD is significantly lower than the maximum allowable concentration of Hg^2+^ (10^−8^ M) in drinking water, as defined by the US Environmental Protection Agency (EPA) [68]. A comparison of the LOD of TiOPc/MoS_2_ with previously reported SERS substrates, shown in Table S6, highlights its superiority among the reported SERS substrates. Figure 5e illustrates a line of calibration demonstrating a linear correlation between Raman intensity and the concentration of Hg^2+^ ions (R^2^ ≈ 0.98), thereby confirming quantitative performance. The reproducibility analysis conducted across fifteen distinct substrates, as illustrated in Figure 5f, results in a relative standard deviation of 8.1%, demonstrating consistent responses. The selectivity test depicted in Figure S14b reveals a prominent Raman signal for Hg^2+^ ions, whereas the other metal ions (Cu^2+^, Pb^2+^, Cd^2+^, Ca^2+^, Cr^2+^, Mg^2+^, Ni^2+^, Ba^2+^, Ag^+^, Co^2+^, and Mn^2+^) exhibit minimal SERS responses. The results confirm that the 4MPy-TiOPc/MoS_2_ SERS substrate offers a sensitive, selective, and reproducible method for detecting Hg^2+^ ions in water-based contaminations.

## 3. Experimental Section

### 3.1. Materials and Regents

Ammonium tetrathiomolybdate ((NH_4_)_2_MoS_4_, 99.97%), used for the growth of trilayer MoS_2_, was purchased from Shanghai Yuanye Bio-Technology Co., Ltd., Shanghai, China. Titanium oxyphthalocyanine (TiOPc) was obtained from Shanghai Maklin Biochemical Technology Co., Ltd., Shanghai, China. Methylene Blue (MB), R6G, Malachite Green (MG), and 4-mercaptopyridine (4-MPy) were purchased from Sinopharm Chemical Reagent Co., Ltd., Shanghai, China. Standard aqueous solutions of Hg^2+^, Cu^2+^, Pb^2+^, Cd^2+^, Ca^2+^, Cr^3+^, Mg^2+^, Ni^2+^, Ba^2+^, Ag^2+^, Co^2+^, and Mn^2+^ were supplied by Tongroma Technology (Beijing) Co., Ltd., Beijing, China.

### 3.2. MoS_2_ Preparation

The synthesis of two-dimensional (2D) MoS_2_ flakes was achieved by a chemical vapor deposition (CVD) technique, which is already reported in the previous literature [2,25,31]. To make the precursor solution, ammonium tetrathiomolybdate ((NH_4_)_2_MoS_4_, 99.95% pure) was dissolved in ethylene glycol at a concentration of 12.5 mg/mL. Silicon wafers with a 300 nm SiO_2_ layer were used as substrates. They were first cleaned with acetone, ethanol, and deionized water for 30 min each. The precursor solution was subsequently applied to the substrates by a spin-coating approach, including a two-step process: an initial 5-s spin at 500 rpm, followed by a 15-s spin at 3000 rpm. The coated wafers were then positioned in a quartz tube and underwent thermal annealing at 550 °C for 90 min in a hydrogen environment. This procedure led to the formation of 2D MoS_2_ flakes on the substrate surface.

### 3.3. Fabrication of TiOPc/MoS_2_ Heterostructure

TiOPc powder, without further purification, was strategically placed into an evacuated quartz furnace tube. The fabrication of nanoparticles was conducted by physical vapor deposition (PVD). The MoS_2_/SiO_2_/Si substrate was positioned 10 cm away from the source material at the far end of the furnace. The deposition process was conducted in 7 min at a base pressure of 10^–3^ Torr, followed by natural cooling to ambient temperature.

### 3.4. SERS Experiments

The SERS efficacy of TiOPc/MoS_2_ nanocomposites was evaluated utilizing methylene blue (MB) and R6G as probe molecules. MB concentrations varied from 10^–7^ to 10^–14^ M, whereas R6G concentrations ranged from 10^–7^ to 10^–13^ M. Aqueous solutions of R6G, MB, and malachite green (MG) at different concentrations were obtained via serial dilution. The substrates were subsequently employed to detect MG within the concentration range of 10^–7^ to 10^–12^ M. For SERS analysis, a 2 μL droplet of each molecule solution was deposited onto the substrates and left to air-dry. Spectral data were acquired by averaging measurements from fifteen randomly chosen points on the sample. The SERS performance of the TiOPc/MoS_2_ substrates was assessed by calculating the enhancement factor (EF) using a method described in the Section S1 of Supporting Information.

### 3.5. Density Functional Calculations

Theoretical calculations of the 2D-MoS_2_ with TiOPc were performed using the QuantumATK 2019 software package with density functional theory (DFT). The exchange-correlation functional is approximated by the generalized gradient approximation (GGA) with the Perdew–Burke–Ernzerhof (PBE) functional. Core electrons of all constituent atoms were represented by FHI norm-conserving pseudopotentials, while valence electrons were expanded using a double-zeta polarized (DZP) basis set to ensure uniform numerical consistency across the system. A mesh cutoff of 75 hartree is adopted for the density of the real space grids. The counterpoise-corrected (CP) adsorption energy is calculated to mitigate the basis set superposition error (BSSE). In geometry optimization, the maximum residual force on each atom is less than 0.04 eV/Å. The total energy tolerance is set to 10^−6^ eV. Moreover, a 20 Å vacuum layer in the z direction is sufficient to ensure no periodic mirror interaction.

### 3.6. Electromagnetic Wave, Frequency Domain (EWFD) Simulation

The simulation of electromagnetic “hot spots” produced by TiOPc nanoparticles on the MoS_2_ surface was conducted using the electromagnetic wave frequency domain (EWFD) model in COMSOL 6.0. Simulation parameters were obtained from SEM measurements of the TiOPc/MoS_2_ configuration. Periodic boundary conditions and completely matched layers were implemented along the X, Y, and Z axes. The system was illuminated along the *Z*-axis using a 633 nm light source, and an ultra-fine mesh size was utilized across all axes to guarantee precision. The refractive indices for SiO_2_, MoS_2_, and TiOPc were 1.4, 1.9, and 2.7, respectively, according to prior research [25,69].

### 3.7. Characterization

The morphology of the synthesized nanostructures was analyzed using SEM (Zeiss, Oberkochen, Germany, Sigma 500, SDNU) with EDS at an accelerating voltage of 7 kV. The particle size and interparticle spacing of TiOPc nanoparticles were evaluated using ImageJ software 1.54s. The crystalline structure was determined by X-ray diffraction (Bruker, D8-Advance, HUASUAN TECHNOLOGY, Shenzhen, China) with Cu Kα radiation. X-ray photoelectron spectroscopy (XPS) using a Thermo Scientific Escalab 250 Xi (HUASAN TECNOLOGY, Shenzhen, China) was utilized to assess elemental composition and chemical states, while ultraviolet photoelectron spectroscopy (UPS) was performed using a Thermo Fisher Scientific Escalab Xi^+^ (HUASAN TECNOLOGY, China) system to examine valence band states. Photoluminescence (PL), Raman, and SERS measurements were conducted utilizing a laser Raman spectrometer (Horiba, HR Evolution, SDNU) including a 600 g/mm diffraction grating, with two accumulations and a collection duration of 4 s. A 325 nm laser (50 mW) was utilized for photoluminescence, whilst 532 nm and 633 nm lasers (0.48 mW) were employed for Raman and SERS to mitigate heat effects. Raman and PL studies were performed under similar conditions: room temperature, 50× magnification, and a laser spot size of 2 μm. UV–vis absorption spectra were obtained with a double-beam spectrophotometer (Purkinje, TU-1900, SDNU).

### 3.8. Photodegradation Experiment

The photocatalytic efficacy of the TiOPc/MoS_2_ composite was assessed by observing the photodegradation of MB dye under xenon lamp illumination. The Xenon lamp, functioning at 30 V, delivered a calibrated light intensity of roughly 200 mW·cm^−2^, with a distance of 4 cm between the lamp and the sample surface. The substate was subjected to the light source for different time periods to investigate the degradation kinetics.

### 3.9. SERS Detection of Hg^2+^ Ions in Water

Aqueous solutions of mercury ions (Hg^2+^) were utilized as target analytes for the practical assessment of water contamination. This study utilized 4-mercaptopyridine (4MPy) as a Raman reporter molecule for the SERS-based detection of Hg^2+^ ions [64]. A 10^−2^ M stock solution of 4MPy was obtained by dissolving 0.11116 g of 4MPy in ethanol, followed by repeated dilutions to achieve the desired lower concentrations. The TiOPc/MoS_2_ substrates were submerged in the 4MPy solution for 30 min to facilitate molecular adsorption, thereafter rinsed thrice with deionized water to eliminate weakly bound molecules, and dried under ambient conditions. An aqueous stock solution of Hg^2+^, kept at 4 °C, was diluted to create a range of concentrations from 10^−5^ to 10^−11^ M. For SERS measurements, 5 µL of the Hg^2+^ solution was applied to the 4MPy-functionalized TiOPc/MoS_2_ substrate to assess its sensing performance.

## 4. Conclusions

This study establishes a new design in organic semiconductor SERS substrate by transcending the limitations of static band engineering through HCGR-driven kinetic modulation. The TiOPc/MoS_2_ heterostructure exemplifies this strategy, where the intrinsically high HCGR of TiOPc acts as a “kinetic pump” to sustain non-equilibrium carrier populations, thereby enabling ECT at the interface. The platform achieves breakthrough SERS performance, with enhancement factors of 1.56 × 10^7^ (MB) and 1.61 × 10^7^ (R6G), detection limits of 10^−14^ M and 10^−13^ M, respectively, reproducibility (RSD = 7.2%), and 60-day stability. Critically, ECT accelerates photodegradation kinetics, achieving complete MB decomposition within 80 min under UV irradiation while retaining SERS functionality. Environmental monitoring capabilities are demonstrated by detecting Hg^2+^ (LOD: 10^−11^ M) and malachite green in tap/lake water (LODs: 10^−12^ M/10^−10^ M) with minimal interference from coexisting ions. This kinetic framework offers transformative potential for real-time industrial wastewater monitoring and process control in chemical engineering. Future work will optimize carrier injection pathways and extend the HCGR/ECT strategy to other 2D materials for high-throughput sensing platforms.

## Data Availability

The data supporting the findings of this study are available within the article and its Supplementary Materials.

## Supplementary Materials

The following supporting information can be downloaded at: https://www.mdpi.com/article/10.3390/molecules31101644/s1, Calculation method of EF S1: Figure S1: SEM images of the synthesized nanostructures at different growth times; Figure S2: Size distribution of nanoparticles; Figure S3: EDS images; Figure S4: Water contact angle of TiOPc/SiO_2_, MoS_2_/SiO_2_, and TiOPc/MoS_2_; Figure S5: UV absorption spectra and Tauc plots; Figure S6: XPS spectra of MoS_2_ and TiOPc/MoS_2_; Figure S7: PL spectra of SiO_2_ and TiOPc/SiO_2_; Figure S8: UV absorption spectra of R6G and MB solutions; Figure S9: SERS spectra and concentration-dependent analysis of R6G; Figure S10: DFT calculations of the TiOPc/MoS_2_ heterostructure; Figure S11: Local electromagnetic field enhancement of pristine MoS_2_ and TiOPc nanoparticles on MoS_2_; Figure S12: Raman intensity variation of MB over ten repeated photocatalytic degradation cycles; Figure S13: MG concentration-dependent SERS analysis, reproducibility, and repeatability; Figure S14: SERS spectra of 4-MPy and SERS intensities of different ions on TiOPc/MoS_2_; Table S1: Raman mode assignments of TiOPc peaks under 532 and 633 nm excitation; Table S2: Comparison of SERS EF and LOD with previously reported SERS substrates; Table S3: Comparison of photodegradation performance with previously reported SERS substrates; Table S4: Comparison of MG SERS LOD with previously reported SERS substrates; Table S5: Recovery of MG in deionized water and lake water matrices; Table S6: Comparison of Hg^2+^ ion SERS LOD with previously reported SERS substrates. Refs. [2,9,25,37,61,69,70,71,72,73,74,75,76,77,78,79,80,81,82,83,84,85,86,87,88,89,90,91,92,93,94,95,96,97,98,99,100,101,102,103,104,105,106,107,108,109,110,111,112,113,114,115,116,117,118] are cited in the Supplementary materials.
