# Supplementary material for: Efficient Charge Transfer in TiOPc/MoS2 Heterostructure for Dynamically Enhanced SERS Sensing and Photocatalysis
Source: Molecules. 2026 May 13;31(10):1644. doi: 10.3390/molecules31101644 (PMC13210012; doi:10.3390/molecules31101644)
Supplement: Supplementary file 1 [file molecules-31-01644-s001.zip › molecules-4270456-Supplementary.pdf]

molecules

MDPI

Supporting Materials

Efficient Charge Transfer in TiOPc/MoS2 Heterostructure for Dynamically Enhanced SERS Sensing and Photocatalysis

Muhammad Saleem, Min Li, Shuai Qiu, Muhammad Zahid, Min Li, Chengju Guo, Abdur Rahim, Yuzhi Song * and Mei Liu *

School of Physics and Optoelectronics, Shandong Normal University, Jinan 250358, China
* Correspondence: yzsong@sdnu.edu.cn (Y.S.); liumei@sdnu.edu.cn (M.L.)

S1. Calculation Method

1.1. Enhancement Factor (EF)

The enhancement factor (EF) of the TiOPc/MoS_2 nanocomposite was assessed using Equation (1).

$$E_F = \frac{I_{SERS}/N_{SERS}}{I_{bulk}/N_{bulk}} \quad (1)$$

In this expression, I_{SERS} and I_{bulk} denote the Raman intensities of the characteristic peak derived from the SERS spectrum and the standard Raman spectrum of methylene blue (MB) powder on a blank SiO_2 substrate, respectively. N_{SERS} and N_{bulk} represent the quantity of MB molecules stimulated by the laser in the examined volume on the TiOPc/MoS_2 substrate and the SiO_2 substrate, respectively.

$$N_{bulk} = \rho H N_A \times \frac{S_{spot}}{M} \quad (2)$$

The value of N_{bulk} was determined using Equation (2), incorporating the molecular density (ϱ = 1.0 g cm^{-3}) and the molar mass (M = 373.9 g mol^{-1}) of MB. In this context, H signifies the laser penetration depth, while S_{spot} refers to the illuminated laser area. Laser excitations at 532 nm and 633 nm resulted in a penetration depth of 21 μm and a spot area of 2 μm^2. N_A represents Avogadro's constant.

$$N_{SERS} = C_{NR} V N_A \times \frac{S_{spot}}{S_{circle}} \quad (3)$$

In a similar manner, N_{SERS} was derived from Equation (3), with C_{NR} V, and S_{circle} representing the MB concentration (10^{-5} M), solution volume (2 μL), and the spreading area (1 cm^2) on the TiOPc/MoS_2 substrate, respectively. Substituting these parameters into Equation (1) resulted in enhancement factor of TiOPc/SiO_2, MoS_2 /SiO_2, and TiOPc/MoS_2 is 2.5×10^3, 3.8×10^3, and 1.56×10^7, respectively. The EF calculation for additional probe molecules, including rhodamine 6G (R6G), adhered to the same methodology. The density (ϱ) and molar mass (M) of R6G were measured at 1.0 g cm^{-3} and 479.02 g mol^{-1}, respectively, with a concentration of 10^{-6} M under 532 nm laser excitation.

 https://doi.org/10.3390/molecules31101644

S2. Results:

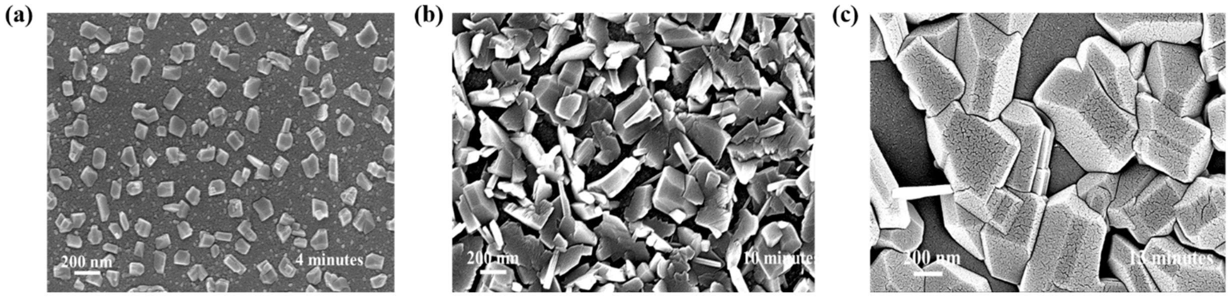

Figure S1. SEM images of the synthesized nanostructures at different growth times: (a) 4 min, (b) 10 min, and (c) 15 min.

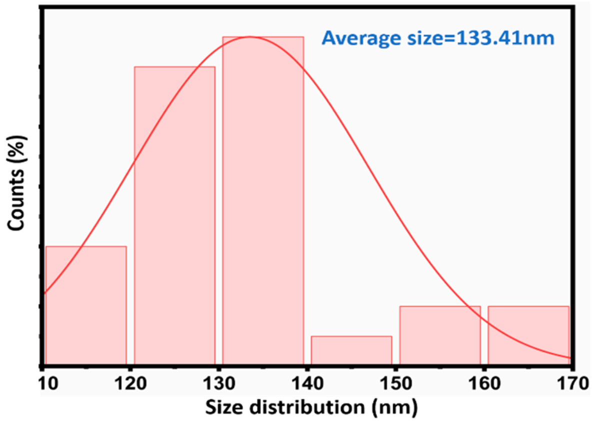

Figure S2. Size distribution of nanoparticles.

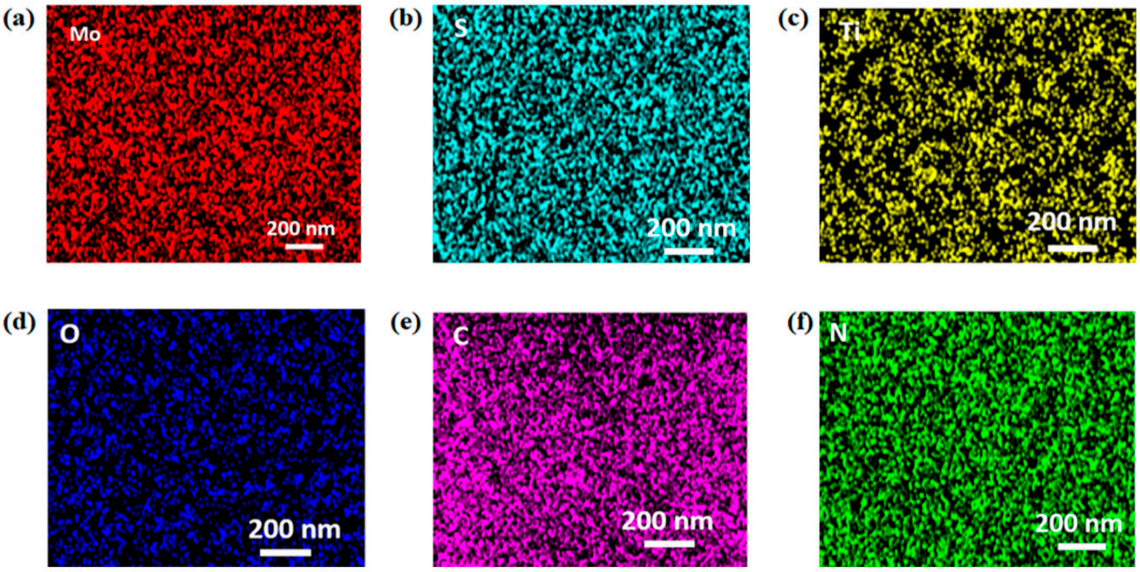

Figure S3. EDS images (a) Mo, (b) S, (c) Ti, (d) O, (e) C and (f) N.

EDS elemental mapping confirms the formation of the TiOPc/MoS_2 heterostructure. The uniform distribution of Mo (Fig. S2a) and S (Fig. S2b) signals verifies the integrity of

the MoS_2 film. The distribution of Ti (Fig. S2c) implies well-deposited TiOPc molecules. The presence of oxygen (Fig. S2d), albeit less pronounced, corresponds with the oxo-ligand in TiOPc. Robust carbon (Fig. S2e) and nitrogen (Fig. S2f) signals correlate to the molecular composition of TiOPc ($C_{32}H_{16}N_8OTi$), with their relative abundance in relation to Ti and O aligning with the molecular structure. The consistent distribution of all elements (Ti, O, C, and N) substantiates the almost homogenous covering of TiOPc on MoS_2 and verifies the successful development of the TiOPc/MoS_2 heterostructure without modifying the MoS_2 stoichiometry.

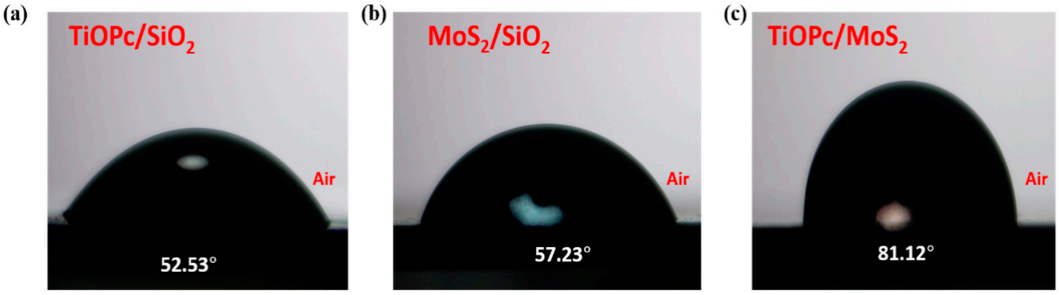

Figure S4. water contact angle of (a) TiOPc/SiO_2, (b) MoS_2/ SiO_2 and TiOPc/MoS_2,.

The wettability of different SERS substrates was assessed by measuring their water contact angles, as illustrated in Fig. S3. The pristine TiOPc had a contact angle of 52.53° (Fig. S3a), signifying a moderately hydrophilic surface that permitted water droplets to disperse well, thereby promoting the homogeneous adsorption of analyte molecules. In contrast, pristine MoS_2 exhibited a somewhat elevated contact angle of 57.23° (Fig. S3b), indicating a balance between its polar edge sites and relatively inert basal planes, leading to intermediate wettability. The deposition of TiOPc onto MoS_2 resulted in a substantial increase in the contact angle to 81.12° (Fig. S3c), indicating a shift towards hydrophobic characteristics. This change is due to the π-conjugated macrocyclic structure of TiOPc and the surface covering of MoS_2, which together reduce the polar interactions with water. The enhanced hydrophobicity of the TiOPc/MoS_2 heterostructure is crucial for SERS, since it promotes the concentration of analytes in reduced droplet regions during solvent evaporation. This process increases the density of electromagnetic hotspots, leading to a more significant augmentation of the Raman signal [1, 2].

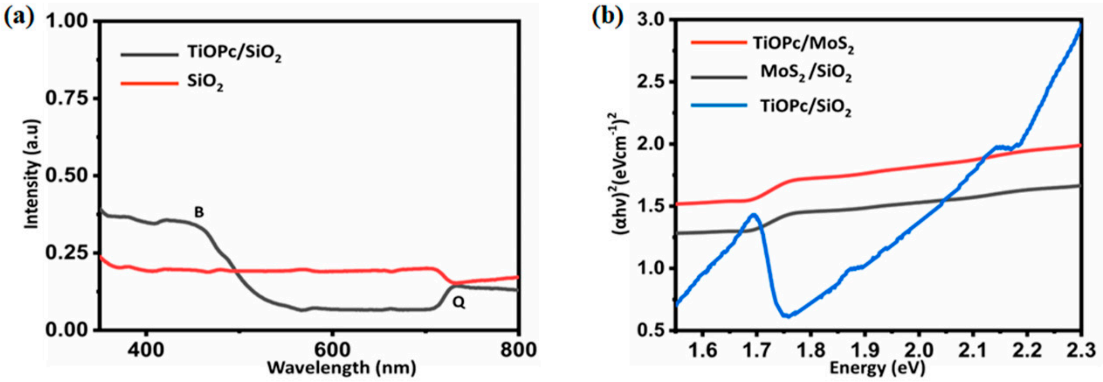

Figure S5. (a) UV absorption spectra of SiO_2 and TiOPc/SiO_2, and (b) Tauq plot of band gap of MoS_2/SiO_2, TiOPc/SiO_2 and TiOPc/MoS_2.

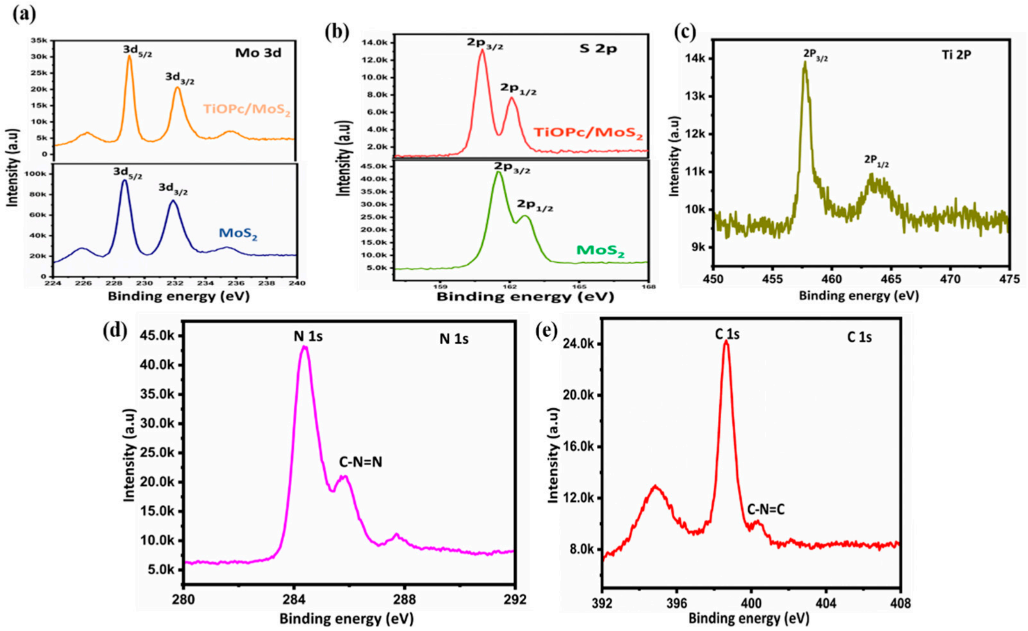

Figure S6. XPS spectra of MoS_2 and TiOPc/MoS_2 (a) Mo 3d, (b) S 2p, (c) Ti 2p, (d) N 1s, and (e) C 1s.

XPS was employed to examine the chemical bonding states and electronic interactions at the interface of titanium phthalocyanine (TiOPc) and molybdenum disulfide (MoS_2). The Mo 3d spectrum of virgin MoS_2 (Fig. S6a) exhibits peaks at 228.68 eV (Mo $3d_{5/2}$) and 231.88 eV (Mo $3d_{3/2}$), signifying Mo in the +4-oxidation state. The S 2p spectra (Fig. S6b) exhibits peaks at 161.35 eV (S $2p_{3/2}$) and 162.68 eV (S $2p_{1/2}$), thereby affirming the existence of S^{2-} within the MoS_2 lattice. Following TiOPc deposition, both Mo and S core-level peaks exhibit a shift to lower binding energies, indicating interfacial CT. The Mo $3d_{5/2}$ peak shifts from 228.68 eV to 229 eV (a 0.4-eV shift), while the Mo $3d_{3/2}$ peak transitions from 231.88 eV to 231.38 eV (a 0.5 eV shift). The S $2p_{3/2}$ peak shifts from 161.35 eV to 161.85 eV (a 0.5 eV shift), while the S $2p_{1/2}$ peak shifts from 162.68 eV to 163.0 eV (a o.32 eV). These shifts signify electron transfer from TiOPc to MoS_2, modifying the electronic environment at the MoS_2 surface, resulting in band bending and the establishment of an interfacial dipole. Alongside the Mo and S core-level shifts, the Ti 2p spectra (Fig. S6c) displays peaks at 463.85 eV (Ti $2p_{3/2}$) and 475.75 eV (Ti $2p_{1/2}$), indicative of Ti in TiOPc. The N 1s spectra (Fig. S6d) exhibit a prominent peak at 394.85 eV, while the C 1s spectrum (Fig. S6e) reveals peaks at 284.35 eV (C-C) and 285.85 eV (C-N), thereby affirming the existence of TiOPc. The detected alterations in the XPS core-level peaks of Mo 3d and S 2p after deposition of TiOPc signify robust electronic interaction at the TiOPc/MoS_2 interface. The displacements in the Mo 3d and S 2p peaks after deposition of TiOPc, indicates the formation of a built-in electric field at the contact. This electric field enables directional charge movement across the junction, an essential characteristic for effective carrier separation and transmission. The interfacial CT processes are essential for facilitating HCGR and UCT in the TiOPc/MoS_2 heterostructure. The XPS data confirm the formation of a heterojunction and provide direct evidence of electronic coupling at the interface, which diminishes the effective barrier for carrier injection into MoS_2 and promotes rapid interfacial CT, as further demonstrated by the enhanced UV-Vis absorption (Fig. 1c) and significantly quenched PL (Fig. 1e) properties of the heterostructure.

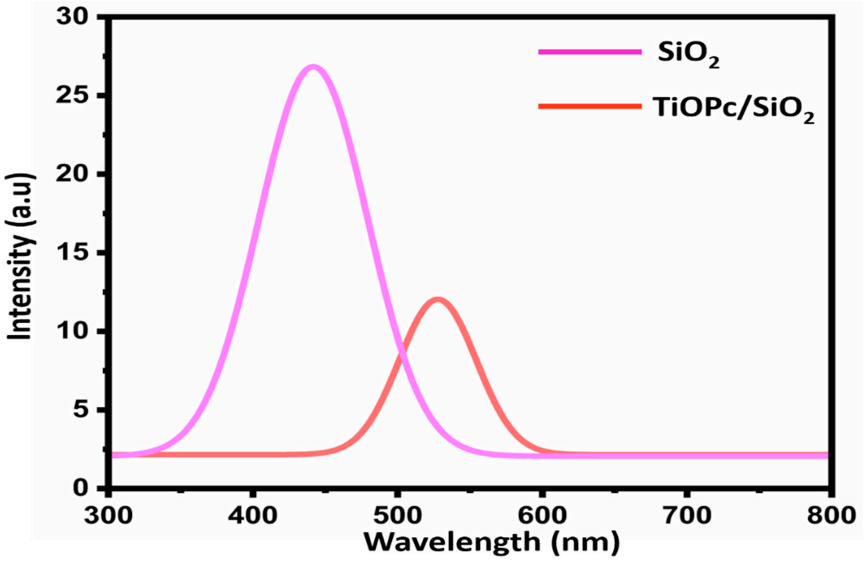

Figure S7. PL spectra of SiO_2 and $TiOPc/SiO_2$.

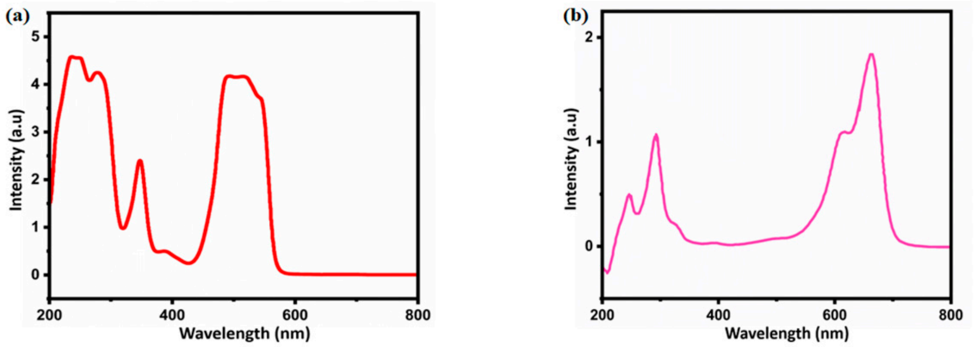

Figure S8. UV absorption spectra (a) R6G solution and (b) MB solution.

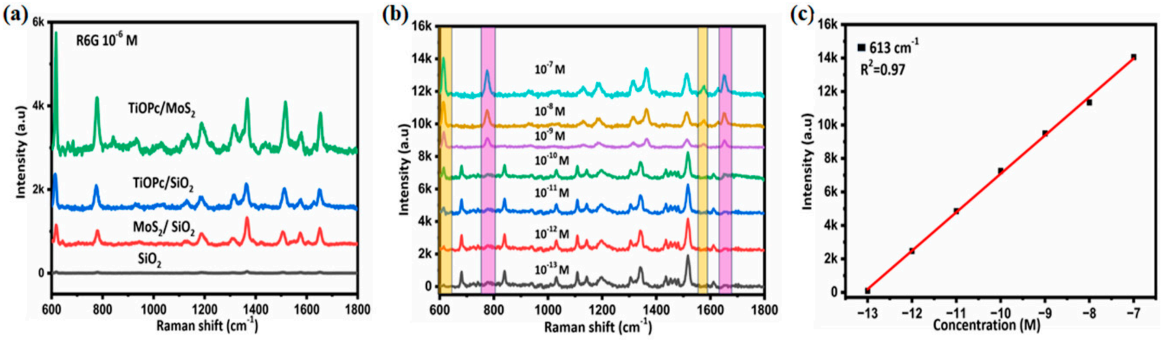

Figure S9. (a) SERS peaks of R6G (10^{-6} M) on different substrates, (b) SERS spectra of R6G on TiOPc/MoS2 from 10^{-7} M to 10^{-13} M, and (c) Correlation between R6G concentrations and the SERS peak intensity at 613 cm^{-1}.

Fig. S8a presents the SERS spectra analysis of R6G on different substrates, which reveal significant variations in peaks intensity and enhancement. The characteristic R6G vibrational modes are observed at 613, 774, 920, 1129, 1316, 1362, 1510, 1577, and 1650 cm^{-1}**,** which correspond respectively to ring deformation, C–H bending, skeletal C–C stretching, C–N stretching, aromatic C–C and C–N stretching, xanthene-ring C–C stretching, and

C=C stretching vibrations [3]. The R6G peaks dominate the spectra, suppressing TiOPc signals due to the formation of a thick R6G layer on the substrate surface and its strong resonant Raman cross-section. The calculated enhancement factor of TiOPc/SiO_2, MoS_2/SiO_2, and TiOPc/MoS_2 is 3.2×10^3, 3.4×10^3, and 1.71×10^7, respectively. Among the tested substrates, the TiOPc/MoS_2 heterostructure exhibits the highest SERS enhancement due to synergetic effect of organic and inorganic components. The comparison of EF and LOD of TiOPc/MoS_2 with previously reported SERS substrate are presented in Table S2, which shows best performance among the reported SERS substrates. This superior SERS performance of the TiOPc/MoS_2 hybrid platform primarily arises from ultrafast charge transfer (UCT), which facilitates efficient charge separation and rapid exciton dissipation, thereby markedly strengthening the chemical enhancement mechanism. In contrast, the contribution of the local electromagnetic field plays a secondary role in the overall SERS enhancement. As R6G concentration decreases from 10^{-7} to 10^{-13} M (Fig. S8b), a transition in spectral dominance from R6G to TiOPc is observed. Below 10^{-9} M, TiOPc bands reappear and gradually dominate the spectrum due to sparse R6G surface coverage. The limit of detection (LOD) analysis for R6G on the TiOPc/MoS_2 substrate shows a strong and stable SERS signal even at ultra-low concentrations, with a linear correlation (R^2=0.99) between Raman intensity and R6G concentration as depicted in Fig. S8c. These findings demonstrate the superior performance of the TiOPc/MoS_2 system as an efficient SERS substrate for ultra-sensitive detection of dye molecules and chemical contaminants.

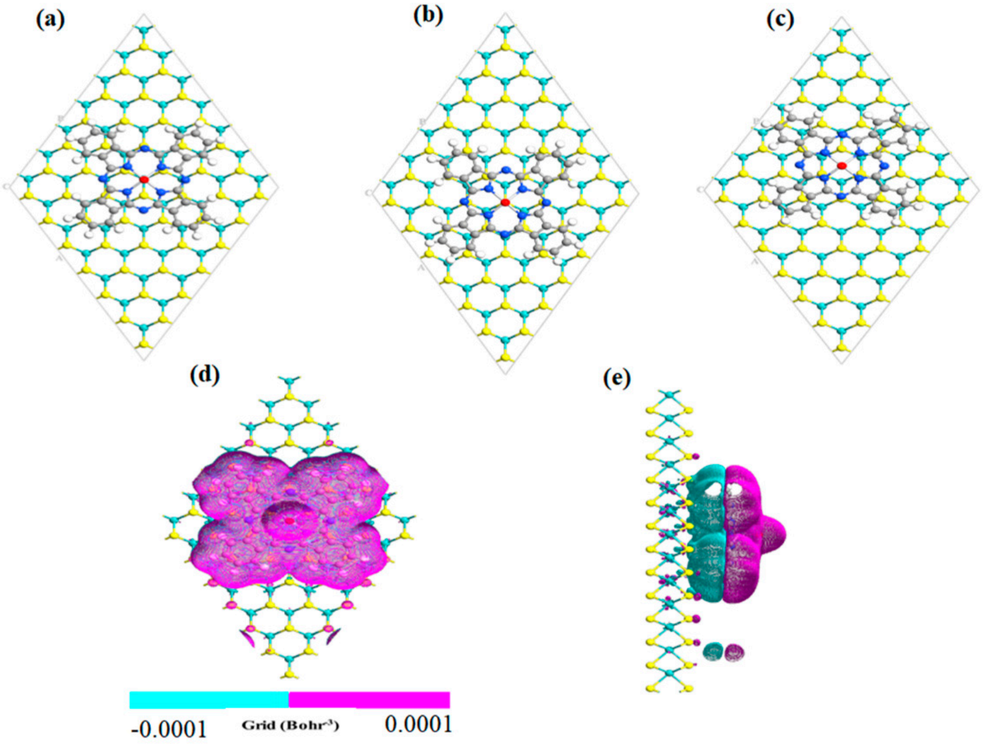

Figure S10. DFT calculations of the TiOPc/MoS_2 heterostructure: (a) TiOPc positioned on top of Mo, (b) TiOPc positioned on top of S, and (c) TiOPc at the hollow site. (d,e) Top and side views of the charge transfer, respectively.

The interaction between the TiOPc ($C_{32}H_{16}N_8OTi$) molecule and the MoS_2 surface was examined at three adsorption sites: Top-Mo, Top-S, and Hollow, illustrated in Fig. S9 (a), (b), and (c), repectively. At the Top-Mo site, where TiOPc adsorbs directly onto

molybdenum (Mo) atoms, the total energy was calculated as -65739.12231 eV, with an adsorption energy of -2.45547 eV. This notably negative adsorption energy indicates a strong interaction, making the Top-Mo site the most favorable for SERS. Such strong adsorption is crucial for enhancing the Raman signal, as it ensures effective molecular binding that amplifies Raman scattering. At the Top-S site (Fig. S9b), where TiOPc adsorbs onto sulfur (S) atoms, the total energy measured was -65736.78127 eV, which is less negative than at the Top-Mo site. The absence of reported adsorption energy suggests a weaker interaction here, likely resulting in less effective Raman signal enhancement and rendering the Top-S site less optimal for SERS applications. Similarly, the Hollow site (Fig. S9c), where TiOPc adsorbs in a hollow region of the MoS_2 lattice, showed a total energy of -65736.77719 eV. Like the Top-S site, no adsorption energy was reported, indicating a weak interaction. Consequently, the Hollow site also offers limited potential for SERS enhancement compared to the Top-Mo site. Charge transfer between TiOPc and MoS_2 is a key factor influencing the SERS effect, as it alters local electron density and thereby enhances Raman scattering. Charge transfer was analyzed under three conditions: Combination, Independence, and Transfer, with Fig. S9 (d) and (e) depicting the process from top and side views, respectively. In the Combination scenario, representing strong interaction between TiOPc and MoS_2, the charge on MoS_2 was 882.047 eV, while TiOPc held 193.9536 eV. This significant charge redistribution demonstrates strong electronic coupling, essential for maximizing SERS enhancement. The pronounced charge transfer here confirms the effectiveness of this interaction in boosting the Raman signal. Under the Independence scenario, where the electronic properties of MoS_2 and TiOPc are considered largely uncoupled, charges were 882.0006 eV on MoS_2 and 194 eV on TiOPc. The minimal variation in charge distribution reflects a weaker interaction, which would correspond to a diminished SERS effect. In the Transfer scenario, MoS_2 experienced a positive charge transfer of +0.0464 eV, while TiOPc showed a corresponding negative transfer of -0.0464 eV. This electron exchange highlights the nature of molecular interaction, as charge transfer modifies the local surface charge distribution, further enhancing the Raman signal and contributing to the SERS effect.

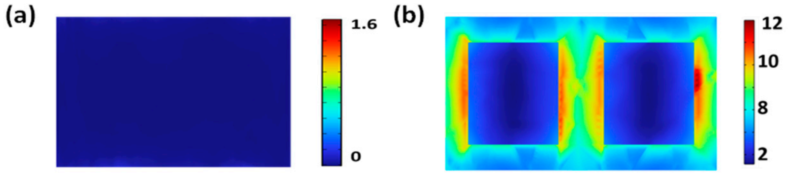

Figure S11. The local electromagnetic field enhancement of (a) pristine MoS_2 and (b) TiOPc nanoparticles on MoS_2 surface.

COMSOL simulations using 633 nm excitation reveal that TiOPc nanoparticles on MoS_2 show significant localized surface plasmon resonance (LSPR) effects, as depicted in Fig. S10b, while pristine MoS_2 (Fig. S10a) does not exhibit any enhancement. The electric field demonstrates considerable concentration at the interparticle gaps, leading to hot spots that significantly amplify the SERS signals of probe molecules. This LSPR effect arises from the resonance between the 633 nm excitation and the Q-band (Fig. S4a) absorption of TiOPc, resulting in collective oscillations of delocalized π-electrons within the nanoparticles. The electromagnetic field's strength and distribution are affected by various factors, such as gap size, particle shape, particle size, and refractive index. The close proximity of nanoparticles is crucial for concentrating the electromagnetic field, leading to enhanced field strengths in these localized areas. The morphology and

dimensions of particles also significantly affect the distribution of electromagnetic fields. Additionally, the refractive index of the surrounding medium significantly influences the extent of field amplification, with higher refractive indices typically enhancing the LSPR effect. The large planar conjugated structure of TiOPc, combined with its high carrier generation rate (HCGR), plays a crucial role in enhancing the localized electromagnetic field due to its structural characteristics. The planar conjugated structure of TiOPc enhances its interaction with light through a delocalized π-electron system, which amplifies the local electromagnetic field, particularly at interparticle gaps where the SERS signals of probe molecules are significantly enhanced. This planar structure further improves the alignment and packing of TiOPc on the MoS_2 substrate, enhancing the localized electromagnetic field and optimizing the SERS signal of the probe molecule. The HCGR of TiOPc is critical for improving the electromagnetic field in these localized areas. When illuminated to light, TiOPc generates a significant number of charge carriers (electrons and holes) that interact with the electromagnetic field, thus amplifying the localized electric fields around the nanoparticles. Unlike metals that contain a high density of free electrons, resulting in significant localized electromagnetic field enhancement, TiOPc, as a semiconductor, has a limited number of free electrons. In semiconductors, charge transfer serves as the principal mechanism for enhancing the SERS signal. The charge transfer mechanism, defined by the transfer of electrons between energy states or molecules, is more influential in the enhancement factor than electromagnetic effects. Therefore, the experimental enhancement factor (EF) of 10^7 exceeds the COMSOL simulated value, mainly due to ultrafast charge transfer linked to the semiconducting characteristics of $TiOPc/MoS_2$.

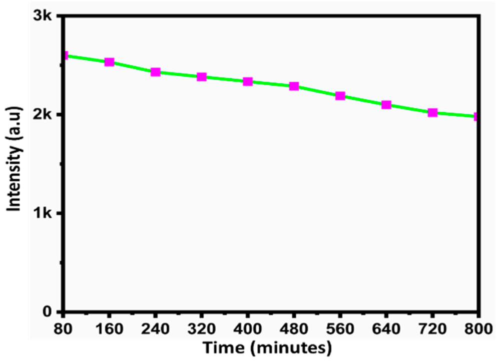

Figure S12. Raman intensity variation of MB over ten repeated photocatalytic degradation cycles under UV irradiation.

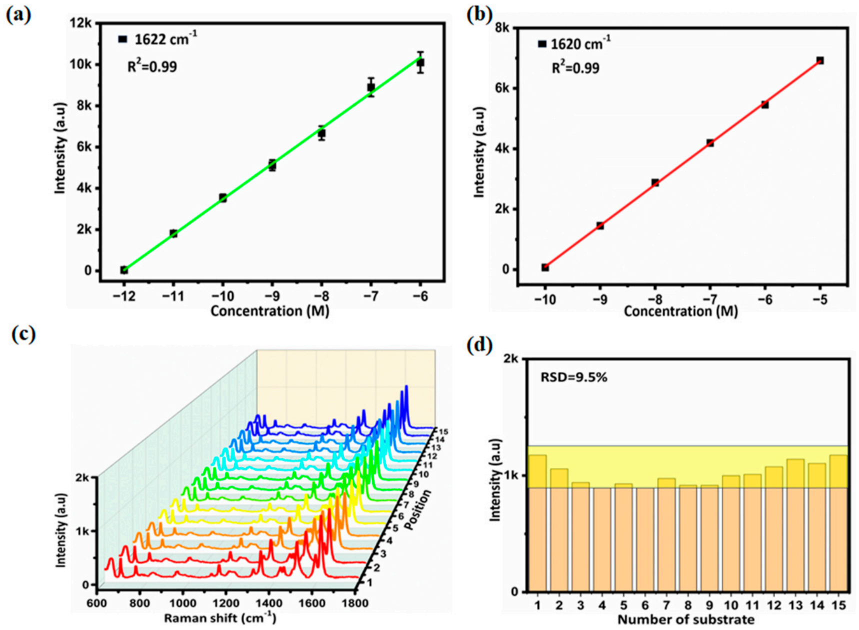

Figure S13. (a) Correlation between MG concentration and SERS peak intensity at 1622 cm⁻¹. (b) Correlation between MG concentration and SERS peak intensity in lake water at 1620 cm⁻¹. (c) SERS reproducibility of MB (10^{-5} M) on the TiOPc/MoS_2 substrate, measured at ten random locations. (d) SERS repeatability of the 1620 cm⁻¹ peak for MG in lake water.

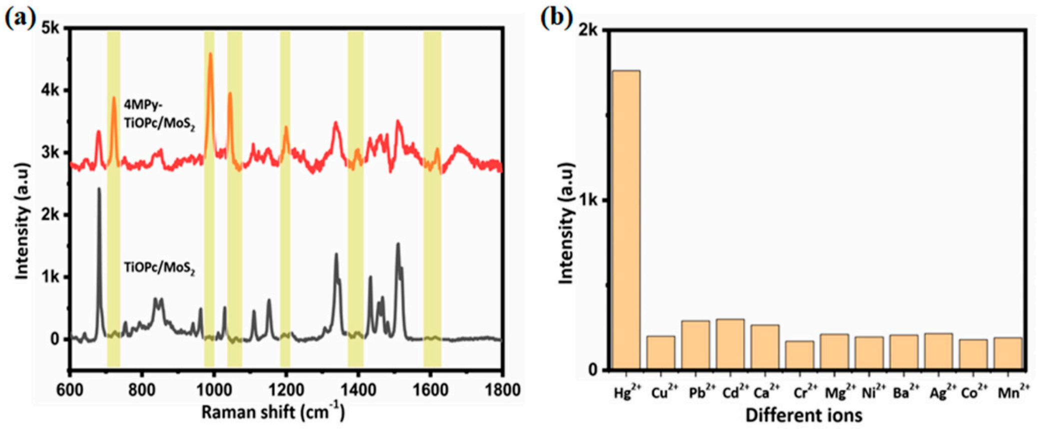

Figure S14. (a) SERS spectra of 4-MPy on TiOPc/MoS_2 and (b) SERS intensities of different ions on TiOPc/MoS_2.

Table S1. Raman modes assignment of TiOPc peaks on various substrate under excitation wavelength 532 and 633 nm.

S.NO	TiOPc/SiO₂ under 532 nm	TiOPc/MoS₂ under 532 nm	TiOPc/SiO₂ under 633 nm	TiOPc/MoS₂ under 633 nm	Raman mode assignment
1	682	680	678	674	Cα-Nα-Cα wagging, C-H out-of-plane
2	725	725	-	-	Cα-Nα-Cα wagging, C-H out-of-plane
3	754	754	750	746	C-H out-of-plane
4	-	774	-	-	Nα-Ti-Nα stretching symmetrical, benzene stretching
5	791	793	-	-	Nα-Ti-Nα stretching asymmetrical, benzene stretching

6	840	835	-	828	Nα-Ti-Nα stretching symmetrical, benzene stretching
7	852	854	-	-	C-H out-of-plane
8	943	941	940	946	C-H out-of-plane
9	963	963	-	-	C-H out-of-plane
10	1031	1029	-	-	Ti-O stretching out-of-plane
11	1110	1110	1105	1107	Cα-Cβ-Cγ stretching asymmetrical 241 247 A1g Isoindole in-phase 1110 1102 A1g Benzene stretching, Nα-
12	1152	1152	1150	1140	Benzene stretching, Nα-Ti-Nα stretching symmetrical, C-C-H
13	-	-	1190	1198	Cα-Nα-Cα stretching asymmetrical, C-C-H
13	-	-	1208	1218	C-H bend
13	1305	-	1304	1304	Cα-Nβ-Cα scissoring, C-C-C benzene, Nα-Ti-Nα stretching symmetrical
14	1340	1339	1342	1337	C-C-C benzene
15	1434	1434	1432	1431	Cα-Cβ-Cγ scissoring, C-C-H
16	1454	1466	1450	1446	Cβ-Cγ-Cδ stretching symmetrical, Cα-Nβ-Cα stretching symmetrical, C-C-H
17	1468	1481	-	-	Cα-Nα-Cα scissoring, Cα-Nβ-Cα stretching symmetrical
18	1513	1510	1510	1508	Cα-Nα-Cα scissoring, Cα-Nβ-Cα stretching asymmetrical

Table S2. The comparison of SERS enhancement factor (EF) and limit of detection (LOD) of TiOPc/MoS_2 substrate with previous reported SERS substrate.

S.No	Substrate	Nature of substrate	EF	LOD (M)	Prob molecule	References
1	MoO_2/ZnSe	Semiconductor	1.14×10^3	10^{-8}	MB	[4]
2	TiOPc/MoS_2	Semiconductor	1.56×10^7<br>1.71×10^7	10^{-14}<br>10^{-13}	MB<br>R6G	This work
3	CuPc/MoS_2	Semiconductor		10^{-10}<br>10^{-11}	MB<br>R6G	[5]
4	ZnSe nanowire	Semiconductor	6.92×10^7	10^{-11}	R6G	[6]
5	Fe_3O_4/CoNi LDH/Ag NPs	Metal	5.81×10^8	10^{-10}	MB	[7]
6	Ag/ZnO	Metal/Semiconductor	6.2×10^6	10^{-9}	MB	[8]
7	TiO_2/Ag	Metal/Semiconductor	1.01×10^9	10^{-9}	R6G	[9]
8	D(C_7CO)-BTBT/MoS_2	Semiconductor	1.6×10^8	10^{-12}	MB	[10]
9	Au/GO	Metal/Semiconductor	3.5×10^6	10^{-9}	R6G	[11]
10	MoS_2 QD/rGO	Semiconductor	1.2×10^7	10^{-9}	R6G	[12]
11	Graphene/Ag/TiO_2	Metal/Semiconductor	3.75×10^6	10^{-11}	R6G	[13]
12	Ag/Cu	Metal	1.2×10^7	10^{-10}	R6G	[14]
13	F_4TCNQ/MoS_2	Semiconductor	1.24×10^6	10^{-11}	MB	[15]
14	Fe_3O_4/GO/Ag	Metal/Semiconductor		10^{-9}	MB	[16]
15	PMMA/$CsPbBr_3$/Au		1.24×10^6	10^{-10}	R6G	[17]
16	g-C3N4-modified Au/Ag		9.2×10^7	10^{-11}	R6G	[18]
17	F_4TCNQ/MoS_2	Semiconductor	2.53×10^6	10^{-11}	MB	[15]
18	MoO_3/MoO_2	Semiconductor	1.4×10^5	10^{-10}	MB	[19]

Table S3. Comparison of photodegradation of TiOPc/MoS_2 SERS substrate with previous reported SERS substrates.

S.NO	SERS substrate	Target molecule	Degradation time	Reference
1	TiOPc/MoS_2	MB	80 minutes	This work
2	P3HT@Ag2NCN	MB	8 hours	[20]
3	Ag@ZnO@Bi2WO6	R6G	140 minutes	[21]
4	Ag/TiO2 cotton fabric	PATP	140 minutes	[22]
5	TiO2/Ag NPs	R6G	120 minutes	[9]
6	Ag NPs/TiO2 nanotube arrays	R6G	150 minutes	[23]
7	TiO2@PLDOPA@Ag NPs	MB	5 hours	[24]
8	Ag-decorated/ZnSe nanowires	R6G	120 minutes	[25]
9	MoO2/ZnSe	MB	120 minutes	[4]

Table S4. The comparison of MG SERS limit of detection (LOD) of TiOPc/MoS_2 substrate with previous reported SERS substrate.

S.No	Substrate	Nature	LOD (M)	Reference
1	Ag array/Graphene	Metal/Semiconductor	10^{-11}	[26]
2	Au Nanoparticles	Metal	10^{-7}	
3	Au/Ag	Metal	10^{-9}	[27]
4	TiOPc/MoS_2	Semiconductor	10^{-12}	This work
5	SA-100/MIP		10^{-10}	[28]
6	TiO_2 NFSF/Ti@Ag	Metal/Semiconductor	10^{-11}	[29]
7	100 GSM paper		10^{-9}	[30]
8	Au/Si Nanowire	Metal/Semiconductor	10^{-8}	[31]
9	Fe_3O_4-Au/MIL-100(Fe)		10^{-8}	[32]
10	AgNAs	Metal	10^{-7}	[33]

Table S5. Recovery of MG in deionized water and lake water matrices.

Sample matrix	C_{spiked} (M)	C_{found} (M)	Recovery (%)
	10^{-5}	8.75×10^{-6}	87.5
	10^{-6}	8.60×10^{-7}	86.6
Lake water	10^{-7}	8.37×10^{-8}	83.7
	10^{-8}	7.37×10^{-9}	79.3
	10^{-9}	7.60×10^{-10}	76.0
	10^{-10}	7.80×10^{-11}	78.0
	10^{-5}	9.85×10^{-6}	98.5
	10^{-6}	9.78×10^{-7}	97.8
Deionized water	10^{-7}	9.69×10^{-8}	96.9
	10^{-8}	9.92×10^{-8}	99.2
	10^{-9}	9.87×10^{-9}	98.8
	10^{-10}	9.74×10^{-11}	97.4
	10^{-11}	9.54×10^{-12}	95.4
	10^{-12}	9.63×10^{-13}	96.3

Table S6. The comparison of Hg^{2+} ions SERS limit of detection (LOD) of TiOPc/MoS_2 substrate with previous reported SERS substrate.

S. No	Substrate	Nature	Raman reporter	LOD (M)	Reference
1	Au NPs	Metal	Bismuthiol II	10^{-9}	[34]
2	TiOPc/MoS_2	Semiconductor	4-MPy	10^{-11}	This work
3	Au/Pt NFs	Metal	TMB	10^{-10}	[35]
4	Au NPs	Metal	Phenylboronic acid	10^{-8}	[36]
5	Au gratings	Metal	Thiol–yne	10^{-10}	[37]
6	Ag NPs	Metal	2,3-diaminephenazine	10^{-9}	[38]

7	AuNPs/rGO	Metal/Semiconductor	DNA	10^{-10}	[39]
8	Au NWs	Metallic	Cy_5	10^{-10}	[40]
9	BSA-modified AuNP	Metallic	R6G	10^{-10}	[41]
10	ZnO/Ag nanoarrays	Metal/Semiconductor	RB	10^{-9}	[42]
11	Au NPs array	Metallic	MPy	10^{-8}	[43]
13	Au NPs	Metallic	RB	10^{-9}	[44]
14	Ag_2Te NPs	Metallic	R6G	10^{-9}	[45]
15	Ag NPs	Metallic	4MPy	10^{-10}	[46]
16	Au NPs	Metallic	Diphenyl thiocarbazone	10^{-9}	[47]
17	Au NPs	Metallic	PATP	10^{-9}	[48]
18	Au NPs in Aqueous Solution	Metallic	Rh6G and Rh123	10^{-6}	[49]
19	Au NPs	Metal	2-mercaptoisonicotinic acid	10^{-8}	[50]
20	Fe_3O_4 @ Ag	Magnetic/Metal	DNA	10^{-10}	[51]
21	SiO_2@Au NPs	Metallic	DNA	10^{-8}	[52]
22	Ag–Au NPs	Metallic	R6G	10^{-9}	[53]
23	Au NWs	Metallic	Cy_5	10^{-8}	[54]
24	Cyclodextrin-coated AgNP<br>Cyclodextrin-coated AgNP<br>Cyclodextrin-coated AgNP<br>Cyclodextrin coated Au NPs	Metal core/organic shell	Methimazole	10^{-10}	[55]

References:

1. Sun, C.; Zhang, S.; Wang, J.; Ge, F., Enhancement of SERS performance using hydrophobic or superhydrophobic cotton fabrics. *Surf. Interfaces* **2022,** 28, 101616.
2. Peng, Q.; Wang, N.; Zhu, Y.; Hu, J.; Peng, H.; Li, L.; Zheng, B.; Du, J.; Xiao, D., Hydrophobic AgNPs: one-step synthesis in aqueous solution and their greatly enhanced performance for SERS detection. *J. Mater. Chem. C* **2019,** 7, (34), 10465-10470.
3. Saleem, M.; Rahim, A.; Lyu, B.; Ma, L.; Zahid, M.; Zhang, X.; Li, M.; Song, Y.; Liu, M., Tunable dual-functional SERS-photoluminescence detection enabled by metal phthalocyanine/MoS2 composites: Central metal atom effects. *Appl. Surf. Sci.* **2025,** 712, 164208.
4. Liu, M.; Hu, X.; Zhang, C.; Shafi, M.; Ma, L.; Lv, B.; Rahim, A.; Saleem, M.; Zhao, L., Localized surface plasmon resonance enhanced charge transfer effect in MoO2/ZnSe nanocomposites enabling efficient SERS detection and visible light photocatalytic degradation. *Sens. Actuators B Chem.* **2024,** 398, 134688.
5. Lyu, B.; Lyu, Y.; Ma, L.; Saleem, M.; Rahim, A.; Li, M.; Zhang, X.; Zahid, M.; Liu, M., Fluorescence Quenching SERS Detection: a 2D MoS2 Platform Modified with a Large π-Conjugated Organic Molecule for Bacterial Detection. *Laser Photonics Rev.* **2025,** 19, (14), 2401831.
6. Shafi, M.; Zhou, M.; Duan, P.; Liu, W.; Zhang, W.; Zha, Z.; Gao, J.; Wali, S.; Jiang, S.; Man, B.; Liu, M., Highly sensitive and recyclable surface-enhanced Raman scattering (SERS) substrates based on photocatalytic activity of ZnSe nanowires. *Sens. Actuators B Chem.* **2022,** 356, 131360.
7. Ren, S.; Fu, J.; Liu, G.; Zhang, H.; Wang, B.; Yu, J., Ultrasensitive detection of methylene blue by surface-enhanced Raman scattering (SERS) with Ag nanoparticle-decorated magnetic CoNi layered double hydroxides. *Analytical Methods* **2025,** 17, (5), 1010-1020.
8. Ha Pham, T. T.; Vu, X. H.; Dien, N. D.; Trang, T. T.; Kim Chi, T. T.; Phuong, P. H.; Nghia, N. T., Ag nanoparticles on ZnO nanoplates as a hybrid SERS-active substrate for trace detection of methylene blue. *RSC Advances* **2022,** 12, (13), 7850-7863.
9. Wu, H.-Y.; Lin, H.-C.; Liu, Y.-H.; Chen, K.-L.; Sun, Y.-S.; Hsu, J.-C., Highly Sensitive, Robust, and Recyclable TiO2/AgNP Substrate for SERS Detection. *Molecules* **2022,** 27, (19), 6755.

10. Liu, M.; Zhang, C.; Ou, C.; Hu, X.; Saleem, M.; Rahim, A.; Ma, L.; Lv, B.; Liu, X.; Zhang, W., Heterostructured organic/MoS2 nanowall with synergistic SERS enhancement enabling direct and sensitive detection of contaminants. *Sens. Actuators B Chem.* **2024,** 401, 135007.
11. Chen, Y.-F.; Lee, Y.-C.; Lin, W.-W.; Lu, M.-C.; Yang, Y.-C.; Chiu, C.-W., Application of Nanohybrid Substrates with Layer-by-Layer Self-Assembling Properties to High-Sensitivity Surface-Enhanced Raman Scattering Detection. *ACS Omega* **2024,** 9, (1), 1894-1903.
12. Wu, D.; Chen, J.; Ruan, Y.; Sun, K.; Zhang, K.; Xie, W.; Xie, F.; Zhao, X.; Wang, X., A novel sensitive and stable surface enhanced Raman scattering substrate based on a MoS2 quantum dot/reduced graphene oxide hybrid system. *J. Mater. Chem. C* **2018,** 6, (46), 12547-12554.
13. Wang, Z.; Li, S.; Wang, J.; Shao, Y.; Mei, L., A recyclable graphene/Ag/TiO2 SERS substrate with high stability and reproducibility for detection of dye molecules. *New J Chem* **2022,** 46, (39), 18787-18795.
14. Peng, W.; Xu, Z.; Jia, X.; Liao, Q., A copper foam-based surface-enhanced Raman scattering substrate for glucose detection. *Discov. Nano* **2023,** 18, (1), 7.
15. Liu, M.; Liu, W.; Zhang, W.; Duan, P.; Shafi, M.; Zhang, C.; Hu, X.; Wang, G.; Zhang, W., π-Conjugated Small Organic Molecule-Modified 2D MoS2 with a Charge-Localization Effect Enabling Direct and Sensitive SERS Detection. *ACS Appl. Mater. Interfaces* **2022,** 14, (51), 56975-56985.
16. He, J.; Song, G.; Wang, X.; Zhou, L.; Li, J., Multifunctional magnetic Fe3O4/GO/Ag composite microspheres for SERS detection and catalytic degradation of methylene blue and ciprofloxacin. *J. Alloys Compd.* **2022,** 893, 162226.
17. Wang, X.; Zhai, Y.; Chen, Y.; Zhang, W.; Liang, P.; Zhu, Q.; Zhang, H.; Kang, J.; Wang, L., Highly sensitive and reproducible SERS substrates based on a novel 3D waffle-like PMMA-CsPbBr3-Au ternary film. *Sens. Actuators B Chem.* **2025,** 431, 137434.
18. Liu, X.; Yu, Y.; Xie, T.; Cao, Z.; Li, Z.; Li, Y.; Gu, Y.; Han, C.; Yang, G.; Qu, L., Fabrication of multifunctional g-C_3N_4-modified Au/Ag NRs arrays for ultrasensitive and recyclable SERS detection of bisphenol A residues. *Microchim. Acta* **2024,** 191, (1), 1-12.
19. Ren, P.; Zhou, W.; Ren, X.; Zhang, X.; Sun, B.; Chen, Y.; Zheng, Q.; Li, J.; Zhang, W., Improved surface-enhanced Raman scattering (SERS) sensitivity to molybdenum oxide nanosheets via the lightning rod effect with application in detecting methylene blue. *Nanotechnology* **2020,** 31, (22), 224002.
20. Xu, L.; Wang, T.; Li, X.; Chen, Z., Organic-Inorganic Semiconductor Heterojunction P3HT@Ag2NCN Composite Film as a Recyclable SERS Substrate for Molecule Detection Application. *Chemosensors* **2022,** 10, 469.
21. Korkmaz, I.; Sakir, M.; Sarp, G.; Salem, S.; Torun, I.; Volodkin, D.; Yavuz, E.; Onses, M. S.; Yilmaz, E., Fabrication of superhydrophobic Ag@ZnO@Bi_2WO_6 membrane disc as flexible and photocatalytic active reusable SERS substrate. *J. Mol. Struct.* **2021,** 1223, 129258.
22. Ge, F.; Chen, Y.; Liu, A.; Guang, S.; Cai, Z., Flexible and recyclable SERS substrate fabricated by decorated TiO2 film with Ag NPs on the cotton fabric. *Cellulose* **2019,** 26, (4), 2689-2697.
23. Zhai, H.; Zhu, C.; Wang, X.; Yuan, Y.; Tang, H., Arrays of Ag-nanoparticles decorated TiO2 nanotubes as reusable three-dimensional surface-enhanced Raman scattering substrates for molecule detection. *Front. Chem.* **2022,** 10, 992236.
24. Mazlumoglu, H.; Yilmaz, M., Silver nanoparticle-decorated titanium dioxide nanowire systems via bioinspired poly(l-DOPA) thin film as a surface-enhanced Raman spectroscopy (SERS) platform, and photocatalyst. *Phys. Chem. Chem. Phys.* **2021,** 23, (23), 13396-13404.
25. Shafi, M.; Duan, P.; Liu, W.; Zhang, W.; Zhang, C.; Hu, X.; Liu, C.; Wali, S.; Jiang, S.; Zhang, C.; Man, B.; Liu, M., Recyclable surface-enhanced Raman spectroscopy (SERS) platform fabricated with Ag-decorated ZnSe nanowires and metamaterial. *Sens. Actuators B Chem.* **2023,** 380, 133410.
26. Ouyang, L.; Yao, L.; Zhou, T.; Zhu, L., Accurate SERS detection of malachite green in aquatic products on basis of graphene wrapped flexible sensor. *Anal. Chim. Acta* **2018,** 1027, 83-91.
27. Zhou, X.; Chen, S.; Pan, Y.; Wang, Y.; Xu, N.; Xue, Y.; Wei, X.; Lu, Y., High-Performance Au@Ag Nanorods Substrate for SERS Detection of Malachite Green in Aquatic Products. *Biosensors*, **2023,** 13 (8), 766.
28. Zhang, X.; Luan, L.; Huang, Y.; Yao, M.; Li, P.; Xu, W., Mussel-inspired PDA-based MIP-SERS sensor for the detection of trace MG in environmental water. *Analyst* **2022,** 147, (24), 5701-5709.
29. Jiang, L.; Wei, W.; Liu, S.; Haruna, S.; Zareef, M.; Ahmad, W.; Hassan, M.; Li, H.; Chen, Q., A tailorable and recyclable TiO2 NFSF/Ti@Ag NPs SERS substrate fabricated by a facile method and its applications in prohibited fish drugs detection. *J. Food Meas. Charact.* **2022,** 16, 1-9.
30. Sarma, D.; Biswas; Hatiboruah, D.; Chamuah, N.; Nath, P., 100 GSM paper as an SERS substrate for trace detection of pharmaceutical drugs in an aqueous medium. *J. Phys. D Appl. Phys.* **2022,** 55, (38), 385102.

31. Lee, B.-S.; Lin, D.-Z.; Yen, T.-J., A Low-cost, Highly-stable Surface Enhanced Raman Scattering Substrate by Si Nanowire Arrays Decorated with Au Nanoparticles and Au Backplate. *Sci. Rep.* **2017,** 7, (1), 4604.
32. Lai, H.; Shang, W.; Yun, Y.; Chen, D.; Wu, L.; Xu, F., Uniform arrangement of gold nanoparticles on magnetic core particles with a metal-organic framework shell as a substrate for sensitive and reproducible SERS based assays: Application to the quantitation of Malachite Green and thiram. *Mikrochim. Acta* **2019,** 186, (3), 144.
33. Liu, H.; Hu, Y.; Zhang, Z., Fabricating a Three-Dimensional Surface-Enhanced Raman Scattering Substrate Using Hydrogel-Loaded Freeze-Induced Silver Nanoparticle Aggregates for the Highly Sensitive Detection of Organic Pollutants in Seawater. *Sensors* **2025,** 25, 2575.
34. Duan, J.; Yang, M.; Lai, Y.; Yuan, J.; Zhan, J., A colorimetric and surface-enhanced Raman scattering dual-signal sensor for Hg2+ based on Bismuthiol II-capped gold nanoparticles. *Anal. Chim. Acta* **2012,** 723, 88-93.
35. Bi, N.; Sun, L.; Hu, M.; Song, W.; Xu, J.; Jia, L., Highly sensitive detection of mercury(II) based on colorimetric-SERS dual signal recognition strategy. *Chem. Eng. Sci.* **2025,** 309, 121510.
36. Wang, Q.; Cai, M.; Ma, Y.; Zhang, Y.; Chen, S.; Zhang, S., Phenylboronic Acid-Functionalized Ratiometric Surface-Enhanced Raman Scattering Nanoprobe for Selective Tracking of Hg2+ and CH3Hg+ in Aqueous Media and Living Cells. *Anal. Chem.* **2024,** 96, (33), 13566-13575.
37. Guselnikova, O.; Svorcik, V.; Lyutakov, O.; Chehimi, M. M.; Postnikov, P. S., Preparation of Selective and Reproducible SERS Sensors of Hg^{2+} Ions via a Sunlight-Induced Thiol–Yne Reaction on Gold Gratings. *Sensors*, **2019**, 19, 2110.
38. Qi, G.; Fu, C.; Chen, G.; Xu, S.; Xu, W., Highly sensitive SERS sensor for mercury ions based on the catalytic reaction of mercury ion decorated Ag nanoparticles. *RSC Advances* **2015,** 5, (61), 49759-49764.
39. Ding, X.; Kong, L.; Wang, J.; Fang, F.; Li, D.; Liu, J., Highly Sensitive SERS Detection of Hg2+ Ions in Aqueous Media Using Gold Nanoparticles/Graphene Heterojunctions. *ACS Appl. Mater. Interfaces* **2013,** 5, (15), 7072-7078.
40. Kang, T.; Yoo, S. M.; Kang, M.; Lee, H.; Kim, H.; Lee, S. Y.; Kim, B., Single-step multiplex detection of toxic metal ions by Au nanowires-on-chip sensor using reporter elimination. *Lab Chip* **2012,** 12, (17), 3077-3081.
41. Wang, Y.; Chen, S.; Wei, C.; Xu, M.; Yao, J.; Li, Y.; Deng, A.; Gu, R., A femtogram level competitive immunoassay of mercury(ii) based on surface-enhanced Raman spectroscopy. *Chem. Commun.* **2014,** 50, (65), 9112-9114.
42. Kandjani, A. E.; Ramanathan, R.; Zabara, M.; Sabri, Y. M.; Bhargava, S. K.; Bansal, V., Long-Range Ordered Crystals of 3D Inorganic–Organic Heterojunctions via Colloidal Lithography. *Small Methods* **2019,** 3, (10), 1900080.
43. Li, K.; Liang, A.; Jiang, C.; Li, F.; Liu, Q.; Jiang, Z., A stable and reproducible nanosilver-aggregation-4-mercaptopyridine surface-enhanced Raman scattering probe for rapid determination of trace Hg2+. *Talanta* **2012,** 99, 890-896.
44. Wang, G.; Lim, C.; Chen, L.; Chon, H.; Choo, J.; Hong, J.; deMello, A. J., Surface-enhanced Raman scattering in nanoliter droplets: towards high-sensitivity detection of mercury (II) ions. *Anal. Bioanal. Chem.* **2009,** 394, (7), 1827-1832.
45. Wang, C. W.; Lin, Z. H.; Roy, P.; Chang, H. T., Detection of mercury ions using silver telluride nanoparticles as a substrate and recognition element through surface-enhanced Raman scattering. *Front. Chem.* **2013,** 1, 20.
46. Chen, L.; Qi, N.; Wang, X.; Chen, L.; You, H.; Li, J., Ultrasensitive surface-enhanced Raman scattering nanosensor for mercury ion detection based on functionalized silver nanoparticles. *RSC Advances* **2014,** 4, (29), 15055-15060.
47. Grasseschi, D.; Zamarion, V. M.; Araki, K.; Toma, H. E., Surface enhanced Raman scattering spot tests: a new insight on Feigl's analysis using gold nanoparticles. *Anal. Chem.* **2010,** 82, (22), 9146-9149.
48. Ma, Y.; Liu, H.; Qian, K.; Yang, L.; Liu, J., A displacement principle for mercury detection by optical waveguide and surface enhanced Raman spectroscopy. *J. Colloid Interface Sci.* **2012,** 386, (1), 451-455.
49. Ganbold, E.-O.; Park, J.-H.; Ock, K.; Joo, S. W., Gold Nanoparticle-Based Detection of Hg(II) in an Aqueous Solution: Fluorescence Quenching and Surface-Enhanced Raman Scattering Study. *Bull. Korean Chem. Soc.* **2011,** 32, 519-523.
50. Tan, E.; Yin, P.; Lang, X.; Zhang, H.; Guo, L., A novel surface-enhanced Raman scattering nanosensor for detecting multiple heavy metal ions based on 2-mercaptoisonicotinic acid functionalized gold nanoparticles. *Spectrochim. Acta, Part A* **2012,** 97, 1007-1012.
51. Zuo, F.; Xu, W.; Zhao, A. A SERS approach for rapid detection of Hg^{2+} based on functionalized Fe_3O_4@Ag nanoparticles. *Acta Chim. Sin.* **2019,** 77 (4), 379-386.
52. Lu, Y.; Zhong, J.; Yao, G.; Huang, Q., A label-free SERS approach to quantitative and selective detection of mercury (II) based on DNA aptamer-modified SiO2@Au core/shell nanoparticles. *Sens. Actuators B Chem.* **2018,** 258, 365-372.
53. Li, P.; Liu, H.; Yang, L.; Liu, J., Sensitive and selective SERS probe for Hg(II) detection using aminated ring-close structure of Rhodamine6G. *Talanta* **2013,** 106, 381-387.

54. Kim, H.; Kang, T.; Lee, H.; Ryoo, H.; Yoo, S. M.; Lee, S. Y.; Kim, B., Facile fabrication of multi-targeted and stable biochemical SERS sensors. *Chem. Asian J.* **2013,** 8, (12), 3010-4.
55. Song, C.; Yang, B.; Yang, Y.; Wang, L., SERS-based mercury ion detections: principles, strategies and recent advances. *Sci. China Chem.* **2016,** 59, (1), 16-29.
